# Gene Erosion Can Lead to Gain-of-Function Alleles That Contribute to Bacterial Fitness

**DOI:** 10.1128/mBio.01129-21

**Published:** 2021-07-06

**Authors:** Julien Mortier, Elisa Gayán, Ronald Van Eyken, Oscar Enrique Torres Montaguth, Ladan Khodaparast, Laleh Khodaparast, Bert Houben, Sebastien Carpentier, Frederic Rousseau, Joost Schymkowitz, Abram Aertsen

**Affiliations:** a KU Leuven, Department of Microbial and Molecular Systems, Leuven, Belgium; b KU Leuven, Department of Cellular and Molecular Medicine, Leuven, Belgium; c KU Leuven, SYBIOMA: Facility for Systems Biology Mass Spectrometry, Leuven, Belgium; Stockholm University; Max Planck Institute for Terrestrial Microbiology

**Keywords:** evolution, genetics, heat resistance, protein aggregates

## Abstract

Despite our extensive knowledge of the genetic regulation of heat shock proteins (HSPs), the evolutionary routes that allow bacteria to adaptively tune their HSP levels and corresponding proteostatic robustness have been explored less. In this report, directed evolution experiments using the Escherichia coli model system unexpectedly revealed that seemingly random single mutations in its *tnaA* gene can confer significant heat resistance. Closer examination, however, indicated that these mutations create folding-deficient and aggregation-prone TnaA variants that in turn can endogenously and preemptively trigger HSP expression to cause heat resistance. These findings, importantly, demonstrate that even erosive mutations with disruptive effects on protein structure and functionality can still yield true gain-of-function alleles with a selective advantage in adaptive evolution.

## INTRODUCTION

Proteotoxic stresses are amply encountered in both natural and manmade environments (1–6) and therefore constitute a primordial concern for bacteria. Indeed, conditions such as heat, reactive oxygen species, low pH, and the presence of antimicrobial compounds may cause the misfolding and aggregation of nascent polypeptides and/or existing proteins in the cell (7). To counter these proteotoxic stresses and ensure proper protein homeostasis (proteostasis), bacteria have developed an elaborate protein quality control network that aims to refold or degrade improperly folded and aggregating polypeptides through the action of chaperones and proteases (e.g., DnaK, DnaJ, HtpG, GroEL, Lon, etc.), which are collectively referred to as heat shock proteins (HSPs) (8–10).

In the Escherichia coli model system, the level of HSP expression is mainly controlled by the RpoH (σ^32^) sigma factor, which is itself subjected to extensive transcriptional and translational regulation (11). While the *rpoH* mRNA has a built-in thermosensor that unblocks the ribosome binding site at elevated temperatures, the RpoH protein becomes sequestered by DnaK and GroEL and targeted for degradation by the FtsH protease. When these sequestering chaperones are titrated away by damaged proteins that emerge when the cell encounters proteotoxic stress, RpoH will be released to occupy the RNA polymerase and raise HSP expression (11–14).

Despite extensive knowledge of the genetic regulation of HSPs, the accessible evolutionary routes that allow bacteria to adaptively tune their HSP levels and that account for corresponding intraspecies differences in proteostatic robustness have been explored less. In this context, some E. coli strains were shown to have extended their complement of available HSPs by the lateral acquisition of so-called transmissible loci for protein quality control, thereby massively boosting their resistance against heat and oxidative stress and raising concerns for their increased survival in medical and industrial settings (15, 16). However, the prevalence of these transmissible islands is rather modest (17), and likely, more subtle evolutionary routes for modulating HSP levels exist as well.

In this study, we provide evidence for such an unanticipated adaptive evolutionary route in which E. coli mitigates the impact of proteotoxic stress by mutationally sacrificing the folding fidelity of a single, nonessential, and transiently expressed protein in order to preemptively activate its heat shock response. As such, we demonstrate that erosive mutations with disruptive effects on protein structure can serve as true gain-of-function mutations adaptively raising HSP levels.

## RESULTS

### Adaptation to heat stress selects for mutants with altered PA management.

**FIG 1:**
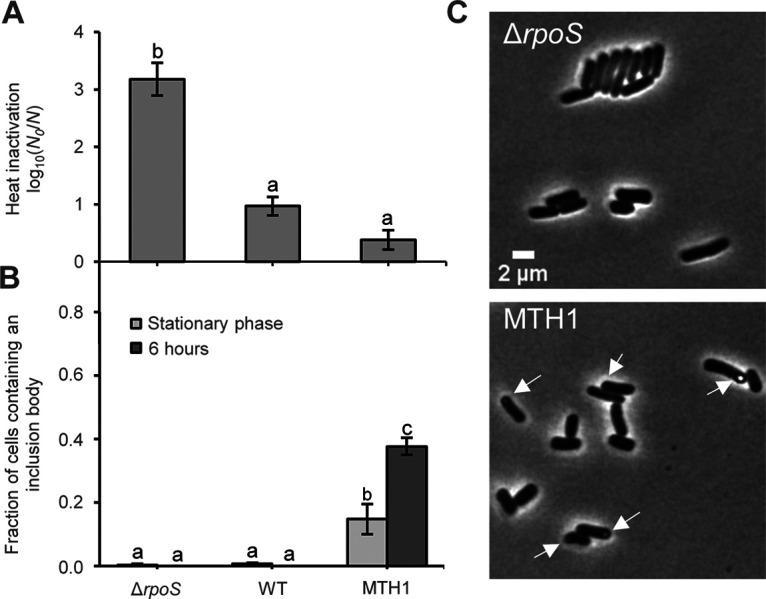
(A) Logarithmic reduction factor after exposure to heat (55°C for 15 min) of TSB-grown stationary-phase cultures of MG1655 wild-type (WT), MG1655 Δ*rpoS*, and a representative heat-resistant mutant (MTH1) derived from MG1655 Δ*rpoS* by directed evolution (i.e., successive cycles of increasingly severe heat shocks with intermittent outgrowth to stationary phase in TSB). Different letters indicate statistically (Tukey HSD *post hoc* test, *P* value ≤ 0.05) significant differences among strains. (B) Fraction of cells that contain an inclusion body visible through phase-contrast microscopy for the indicated strains in TSB-grown stationary-phase populations and 6 h after reinoculating 1/100 in fresh TSB. On average, 93.2 cells were observed per strain and condition per independent experiment, and all sample sizes were between 85 and 110 cells. Different letters indicate statistically significant differences (Tukey HSD *post hoc* test, *P* value ≤ 0.05) among different strains and time points. For panels A and B, the displayed means were determined over three independent experiments, and the error bars indicate the standard error over these experiments. (C) Representative phase contrast images of MG1655 Δ*rpoS* (upper panel) and its evolved heat-resistant MTH1 mutant (lower panel) after stationary-phase growth in TSB. White arrows indicate inclusion bodies. Scale bar corresponds to 2 μm.

**FIG 2:**
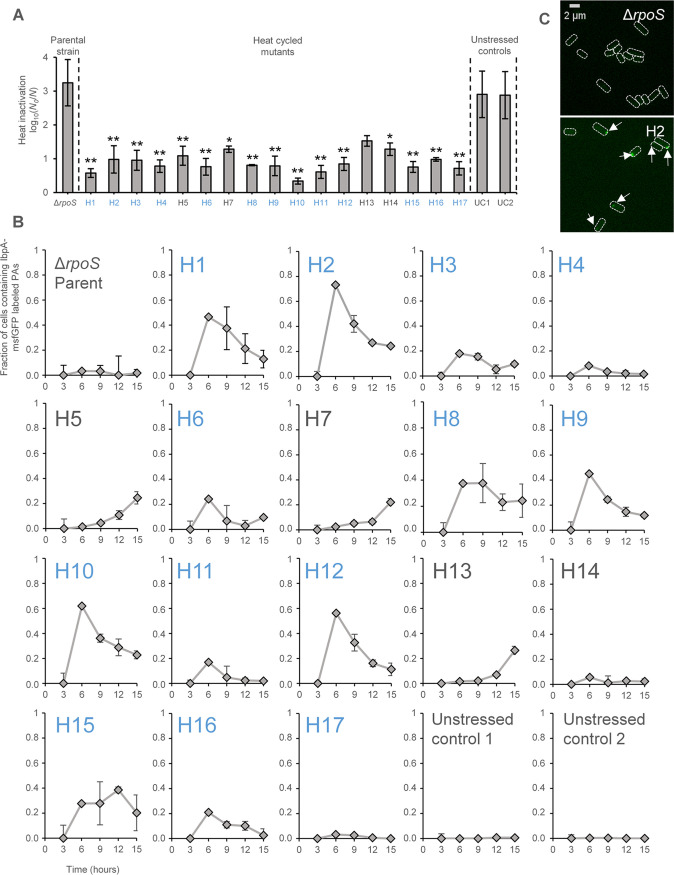
Mutants H1 to H17 were derived as single clones from independent lineages of the MG1655 Δ*rpoS ibpA*-*msfGFP* parental strain that were subjected to successive cycles of increasingly severe heat shocks (ranging from 15 min at 51°C to 55°C with 0.5°C increments) with intermittent outgrowth to stationary phase in TSB. As a control, UC1 and UC2 were derived as single clones from independent lineages of MG1655 Δ*rpoS ibpA*-*msfGFP* that were similarly cycled but without being exposed to heat stress. (A) Logarithmic reduction factor after exposure to heat (55°C for 15 min) of stationary-phase TSB cultures of the parental MG1655 Δ*rpoS ibpA-msfGFP* strain and its indicated derivatives. Asterisks indicate a statistically significant decrease in the logarithmic reduction factor compared to the parental strain (Tukey HSD *post hoc* test; *, *P ≤ *0.05; **, *P* ≤ 0.01). (B) The fraction of cells containing a fluorescent IbpA-msfGFP labeled PA in the parental MG1655 Δ*rpoS ibpA-msfGFP* strain and its indicated derivatives was determined microscopically by sampling a growing TSB culture every 3 h after reinoculating 1/100 from an overnight culture. Out of the 17 heat-selected mutants, 14 (H1, H2, H3, H5, H6, H7, H8, H9, H10, H11, H12, H13, H15, and H16) could be considered as having aberrant cellular PA management compared to the controls on the basis of the IbpA-msfGFP reporter. On average, 100.96 cells were observed per strain and time point per independent experiment, and all sample sizes were between 64 and 148 cells. For panels A and B, the displayed means were determined over three independent experiments, and the error bars indicate the standard error over these experiments. Blue labels indicate mutants with mutations in their *tnaA* allele as determined by whole-genome sequencing. (C) Representative GFP epifluorescence (reporting IbpA-msfGFP expression and localization) images of MG1655 Δ*rpoS ibpA-msfGFP* and one of its heat-selected mutants (H2) after 6 h of exponential growth in TSB. Cell outlines are shown in white, and the white arrows indicate IbpA-msfGFP labeled PAs. Scale bar corresponds to 2 μm.

During preliminary directed evolution experiments, we (i) found that an E. coli MG1655 Δ*rpoS* mutant (i.e., deprived of its σ^S^ general stress response regulator) could readily complement its intrinsic hypersensitivity to heat (Fig. 1A), and (ii) serendipitously noticed through phase-contrast microscopy that some such independently evolved heat resistant MG1655 Δ*rpoS* mutants typically displayed cells bearing a highly refractive polar structure, reminiscent of a protein aggregate (PA) (Fig. 1B and C). To address these initial experimental observations more systematically, we subsequently heat-cycled 17 independent lineages of E. coli MG1655 Δ*rpoS ibpA-msfGFP* (able to fluorescently report on the presence of intracellular PAs through the IbpA-msfGFP reporter [18]), and from each lineage retained one random heat-resistant clone (Fig. 2A). Interestingly, 14 out of these 17 independent heat-resistant mutants (all except H4, H14, and H17) displayed considerably higher fractions of PA-bearing cells (especially toward the early and/or late stationary phase of growth) compared to the parental strain and its two control-cycled derivatives (Fig. 2B and C), thereby consolidating our observation that adaptive evolution toward heat resistance can alter cellular PA management.

### Heat resistance causally stems from gain-of-function TnaA variants.

Surprisingly, whole-genome sequencing of the 17 independent heat-resistant mutants revealed that 13 of them harbored a mutation in the *tnaA* gene (encoding the tryptophanase enzyme; Table 1). Out of these 13 *tnaA* mutants, 11 stood out as having clearly altered cellular PA management, while 2 (H4 and H17) were hardly distinguishable from the parental MG1655 Δ*rpoS ibpA-msfGFP* strain with regard to the IbpA-msfGFP PA reporter (Fig. 2B). Moreover, checking the *tnaA* allele of some of the preliminary heat-cycled mutants, and even of previously reported hydrostatic pressure-cycled mutants (19), revealed another 4 *tnaA* alleles (Table 1). Interestingly, most of these *tnaA* alleles (14 out of 17) were found to be unique and incurred point mutations, premature stop codons, or indels (both in-frame or frameshifting) throughout the *tnaA* open reading frame (Table 1). Moreover, while many *tnaA* mutants were partially or fully compromised in their tryptophanase activity (i.e., the ability to produce indole from tryptophan), some of them displayed no obvious loss-of-function signs (Table 1).

**TABLE 1:** Overview of heat- and pressure-selected mutant *tnaA* alleles found in this study and their activity in producing indole

Strain^*a*^	Allele name	DNA mutation (1,416 bp in total)	Protein mutation (471 aa in total)^*b*^	mol wt (kDa)	Indole activity^*c*^	Source or reference
MTH1	*tnaA^A359P^*	G1075C	A359P	52.8	–	This study
MT2	*tnaA^Q240P^*	A719C	Q240P	52.8	+/–	19
MT3	*tnaA^Δ106^*	In-frame deletion of 318 bp (from position 187 to 504)	In-frame deletion of 106 aa (from position 63 to 168)	40.6	–	19
MT5^1^	*tnaA^V224E^*	T671A	V224E	52.8	+/–	19
H1	*tnaA^ins1^*	3-bp (TAT) insertion between positions 462 and 463	1 in-frame aa (Y) insertion between positions 154 and 155	53.0	+/–	This study
H2	*tnaA^V225E^*	T674A	V225E	52.8	+/–	This study
H3	*tnaA^F131C^*	T392G	F131C	52.7	+/–	This study
H4^2^	*tnaA^Δ31^*	In-frame deletion of 93 bp (from position 838 to 930)	In-frame deletion of 31 aa (from position 280 to 310)	49.3	–	This study
H6	*tnaA^K283E^*	A847G	K283E	52.8	+	This study
H8^3^	*tnaA^A130E^*	C389A	A130E	52.8	+/–	This study
H9^3^	*tnaA^A130E^*	C389A	A130E	52.8	+/–	This study
H10	*tnaA^Δ105+M63S^*	318-bp deletion (from position 188 to 505)	In-frame deletion of 105 aa (from position 64 to 168) + M63S	40.6	+/–	This study
H11	*tnaA^Q119P^*	A356C	Q119P	52.8	+	This study
H12^1^	*tnaA^V224E^*	T671A	V224E	52.8	–	This study
H15	tnaA^G451^*	T1355G (creates an early in-frame stop codon)	Protein truncated after position 451 (G) because of early stop codon	50.4	–	This study
H16^2^	*tnaA^Δ31^*	In-frame deletion of 93 bp (from position 838 to 930)	In-frame deletion of 31 aa (from position 280 to 310)	49.3	–	This study
H17	*tnaA^259fs^*	1-bp (A) deletion at position 777 (creating a frame shift and early stop codon)	Truncated protein with a 23-aa nonsense sequence after position 258 (Y)	32.2	–	This study

a Superscript 1, 2, and 3 indicate independently isolated mutants with identical mutations.

b aa, amino acids.

c +, Enzyme activity comparable to the MG1655 Δ*rpoS* (for MTH1, MT2, MT3, and MT5) or MG1655 Δ*rpoS ibpA-msfGFP* (for H1, H2, H3, H4, H6, H8, H9, H10, H11, H12, H15, H16, and H17) parental strain; +/–, diminished enzyme activity compared to the parental strain; and –, no observable enzyme activity. These designations were based on indole concentrations determined in stationary-phase TSB cultures.

**FIG 3:**
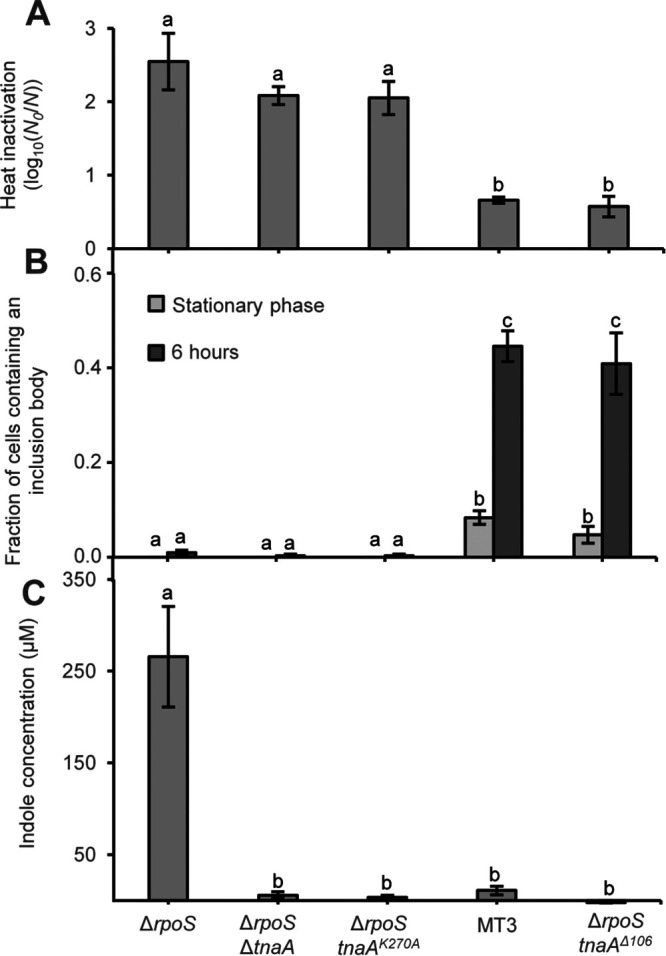
(A) The logarithmic reduction factor after exposure to heat (55°C for 15 min) of stationary-phase TSB cultures of MG1655 Δ*rpoS*, MG1655 Δ*rpoS* Δ*tnaA*, MG1655 Δ*rpoS tnaA^K270A^* (harboring a catalytically inactive TnaA variant), the originally evolved MT3 mutant (harboring the *tnaA^Δ106^* allele), and the reconstructed MG1655 Δ*rpoS tnaA^Δ106^* strain. (B) The fraction of cells that contain an inclusion body visible in phase contrast was determined for the indicated strains after growth in TSB to stationary phase and 6 h after reinoculating 1/100 in fresh TSB medium. On average, 95.9 cells were observed per strain and condition per independent experiment, and all sample sizes were between 80 and 110 cells. (C) Indole concentrations produced by the indicated strains after growth in TSB to stationary phase. For panels A to C, the displayed means were determined over three independent experiments, and the error bars indicate the standard error over these experiments. Within each panel, different letters indicate statistically significant (Tukey HSD *post hoc* test, *P* value ≤ 0.05) differences among different strains and time points.

To infer causality between possible *tnaA* alterations and heat resistance, the wild-type *tnaA* allele (*tnaA^WT^*) of E. coli MG1655 Δ*rpoS* was replaced with either (i) *tnaA^Δ106^* as one of the evolved alleles (coming from mutant MT3; Table 1), (ii) Δ*tnaA* (unable to produce the TnaA protein), or (iii) *tnaA^K270A^* (producing a catalytically compromised TnaA variant [20]) and subsequently heat challenged (Fig. 3A). Interestingly, this revealed not only that the *tnaA^Δ106^* allele itself was indeed causally sufficient to increase heat resistance of MG1655 Δ*rpoS* to the same level as seen in the original MT3 mutant, but also that the Δ*tnaA* and *tnaA^K270A^* alleles failed to affect this phenotype (Fig. 3A), despite sharing an impaired ability to produce indole with the *tnaA^Δ106^* allele (Fig. 3C). Moreover, in contrast to the *tnaA^WT^*, Δ*tnaA*, and *tnaA^K270A^* alleles, implementation of the *tnaA^Δ106^* allele also caused MG1655 Δ*rpoS* to produce PAs to the same level as seen in MT3, indicating that expression of this variant directly or indirectly causes protein aggregation (Fig. 3B). As such, it could be inferred that the complete absence of the TnaA protein or the more subtle loss of its enzymatic function (i.e., indole production) is not sufficient or required to impose heat resistance, indicating that the evolved *tnaA* variants should be considered subtle gain-of-function alleles.

In further consolidation of this causality and the need for *tnaA^Δ106^* to actually become expressed, we also observed that preventing *tnaA^Δ106^* expression by depriving the growth medium of tryptophan (i.e., the inducer of the *tnaCAB* operon [21, 22]) abrogated the heat resistance effect (Fig. S1). Moreover, in tryptone soy broth (TSB) batch cultures, induction of the *tnaCAB* operon naturally occurs toward early stationary phase (when glucose becomes depleted and catabolite repression is alleviated [23–25]), which also corresponds in timing to the rise in PA-containing cells in the selected *tnaA* mutants (Fig. 2B). In contrast, induction of the *tnaCAB* operon already occurs in mid-exponential-phase LB in batch cultures (where amino acids are the main carbon source [26]), and exponential-phase heat resistance in the *tnaA^Δ106^* mutant could accordingly be observed in LB but not in TSB medium (Fig. S2).

10.1128/mBio.01129-21.3 FIG S1 Logarithmic reduction factor after heat exposure (54.5°C for 15 minutes, recovery on TSA) of populations of MG1655 Δ*rpoS*, MG1655 Δ*rpoS tnaA^K270A^*, and MG1655 Δ*rpoS tnaA^Δ106^* grown to stationary phase in AB minimal medium supplemented without (dark grey; no trp) or with (light grey; trp) 1 mM tryptophan. Different lowercase and capital letters indicate statistically significant differences (Bonferroni test, *P* value ≤ 0.05) in inactivation among strains with and without tryptophan induction, respectively. The asterisks indicate statistically significant differences (*t* test, *P* value ≤ 0.05) in the inactivation of each strain with and without tryptophan induction. The displayed means were determined over three independent experiments, and the error bars indicate the standard error over these experiments. Download FIG S1, TIF file, 1.9 MB. Copyright © 2021 Mortier et al. 2021 Mortier et al. https://creativecommons.org/licenses/by/4.0/ This content is distributed under the terms of the Creative Commons Attribution 4.0 International license.

10.1128/mBio.01129-21.4 FIG S2 (A and B) Logarithmic reduction factors after exposure to heat (54°C for 15 minutes, recovery on LB agar) of MG1655 Δ*rpoS*, MG1655 Δ*rpoS tnaA^K270A^*, and MG1655 Δ*rpoS tnaA^Δ106^* grown to exponential phase in (A) LB or (B) TSB. Different letters within panels indicate statistically significant differences (Tukey HSD *post hoc* test, *P* value ≤ 0.05) among strains. The displayed means were determined over three independent experiments, and the error bars indicate the standard error over these experiments. Download FIG S2, TIF file, 2.5 MB. Copyright © 2021 Mortier et al. 2021 Mortier et al. https://creativecommons.org/licenses/by/4.0/ This content is distributed under the terms of the Creative Commons Attribution 4.0 International license.

### Compromised TnaA folding fidelity boosts the level of heat shock proteins.

**FIG 4:**
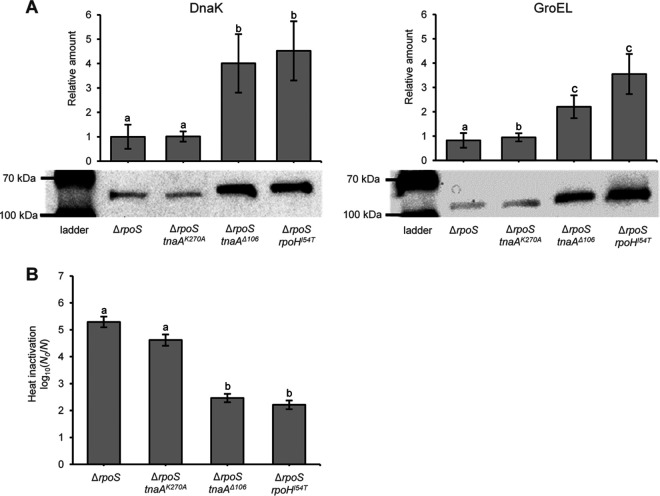
(A) Western blot analysis of DnaK and GroEL expression in stationary-phase cell lysates of MG1655 Δ*rpoS*, MG1655 Δ*rpoS tnaA^K270A^*, MG1655 Δ*rpoS tnaA^Δ^*^106^, and MG1655 Δ*rpoS rpoH^I54T^* grown in TSB. The data display the means of relative amounts of protein (as determined by the density of the bands after background subtraction) compared to the Δ*rpoS* parent determined over four independent experiments, and the error bars indicate the standard error over these experiments. Images below the graphs correspond to a representative gel from one of the independent experiments. (B) Logarithmic reduction factor after heat exposure (56°C for 15 min) of the indicated strains grown to stationary phase in TSB. The displayed means were determined over three independent experiments, and the error bars indicate the standard error over these experiments. Different letters indicate statistically significant differences (Student’s *t* tests followed by Bonferroni correction, *P* value ≤ 0.05) in the relative amount of protein (A) or inactivation (B) among strains.

We subsequently hypothesized that the protein aggregation directly or indirectly inflicted by TnaA variants could lead to activation of the heat shock response, which in turn would provide the cell with increased heat resistance, while simultaneously leading to the emergence of PAs in the cell. Subsequent Western blot analysis indeed revealed that MG1655 Δ*rpoS* equipped with *tnaA^Δ106^* displayed increased levels of heat shock proteins (HSPs) such as DnaK and GroEL compared to MG1655 Δ*rpoS* equipped with either *tnaA^WT^* or *tnaA^K270A^* (Fig. 4A). Moreover, the increase in DnaK and GroEL levels was similar to the included positive control of an MG1655 Δ*rpoS rpoH^I54T^* mutant equipped with a constitutively active variant of the heat shock response sigma factor RpoH (Fig. 4A) (27). In fact, the extent of increased heat resistance of the Δ*rpoS tnaA^Δ106^* mutant coincided with that of the Δ*rpoS rpoH^I54T^* strain (Fig. 4B).

**FIG 5:**
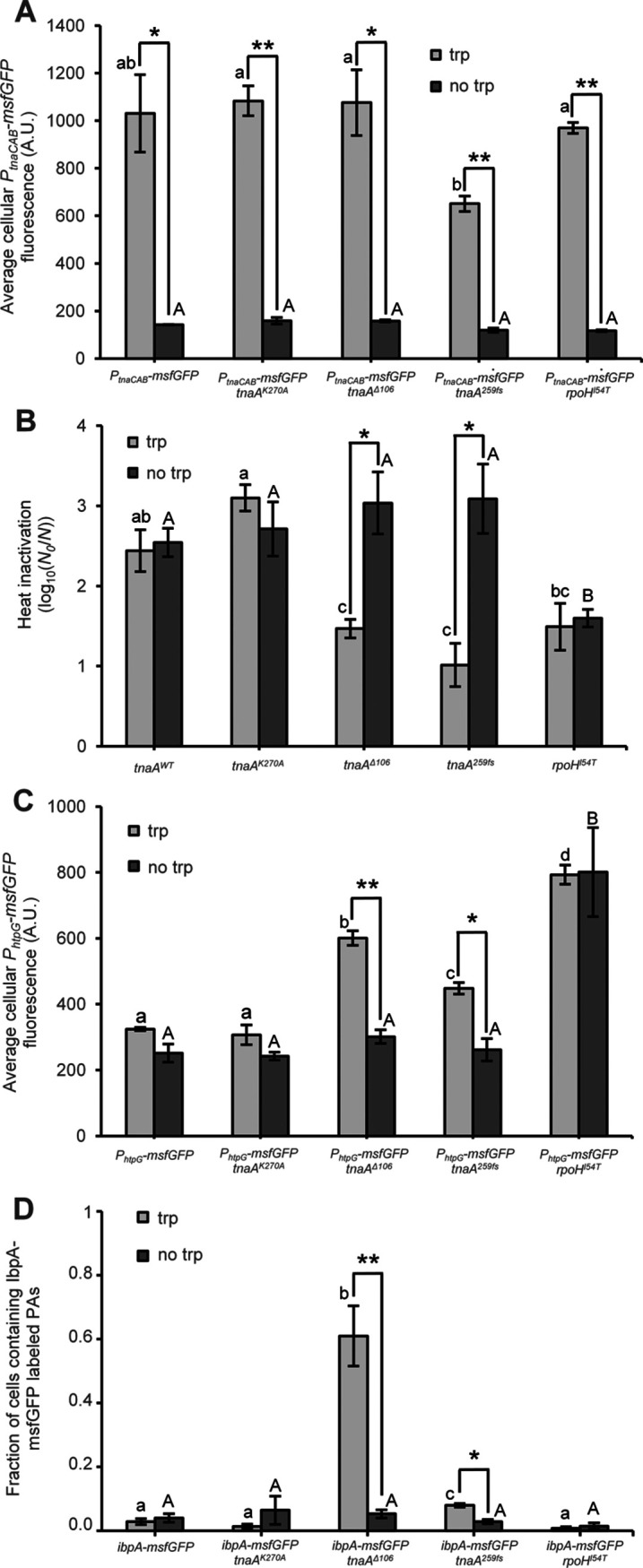
(A) Average cellular fluorescence of AB-grown exponential-phase populations of the MG1655 *P_tnaCAB_-msfGFP* fluorescent reporter (harboring *tnaA^WT^* and *rpoH^WT^*) and its *tnaA^K270A^*, *tnaA^Δ^*^106^, *tnaA^259fs^*, or *rpoH^I54T^* derivatives with (trp; light gray) or without (no trp; dark gray) tryptophan induction. The average number of observed cells per strain and condition per independent experiment was 347.0, and sample sizes were always between 143 and 745 cells. (B) Logarithmic reduction factor after exposure to heat (54.5°C for 15 min, recovery on LB agar) of AB-grown exponential-phase populations of MG1655 (harboring *tnaA^WT^* and *rpoH^WT^*) and its *tnaA^K270A^*, *tnaA^Δ^*^106^, *tnaA^259fs^*, or *rpoH^I54T^* derivatives with (trp; light gray) or without (no trp; dark gray) tryptophan induction. (C) Average cellular fluorescence of AB-grown exponential-phase populations of the MG1655 *P_htpG_-msfGFP* fluorescent reporter (harboring *tnaA^WT^* and *rpoH^WT^*) and its *tnaA^K270A^*, *tnaA^Δ^*^106^, *tnaA^259fs^*, or *rpoH^I54T^* derivatives with (trp; light gray) or without (no trp; dark gray) tryptophan induction. The average number of observed cells per strain and condition per independent experiment was 421.7, and sample sizes were always between 116 and 965 cells. (D) Fraction of cells containing an IbpA-msfGFP labeled PA of AB-grown exponential-phase populations of the MG1655 *ibpA-msfGFP* fluorescent reporter (harboring *tnaA^WT^* and *rpoH^WT^*) and its *tnaA^K270A^*, *tnaA^Δ^*^106^, *tnaA^259fs^*, or *rpoH^I54T^* derivatives with (trp; light gray) or without (no trp; dark gray) tryptophan induction. On average, 91.3 cells were observed per strain and condition per independent experiment, and all sample sizes were between 50 and 106 cells. For all panels, strains were grown to exponential phase in AB medium (i.e., 4 h of growth after 1/1,000 dilution of stationary-phase cultures) with or without addition of 1.25 mM tryptophan for the last 2 h of growth. For all panels, the displayed means were determined over three independent experiments, and the error bars indicate the standard error over these experiments. Asterisks indicate a statistically significant difference for induced compared to noninduced populations of the same strain (Student’s *t* tests followed by Bonferroni correction; *, *P* ≤ 0.05; **, *P* ≤ 0.01), while different lowercase (for tryptophan-induced populations) or capital (for noninduced populations) letters indicate statistically significant differences among strains (Student’s *t* tests followed by Bonferroni correction, *P* value ≤ 0.05).

To more closely and causally link the expression of TnaA variants to induction of PAs, HSPs, and heat resistance, MG1655 (i.e., now bearing its wild type σ^S^ general stress response regulator) was chromosomally equipped with different reporter constructs and different *tnaA* alleles (or the *rpoH^I54T^* allele as an HSP upregulated control), after which corresponding exponential-phase cultures were shortly induced with tryptophan and examined for (i) *tnaCAB* promoter activity (using the *P_tnaCAB_-msfGFP* reporter; Fig. 5A), (ii) heat resistance (Fig. 5B), (iii) HSP expression level (using the RpoH-controlled *P_htpG_-msfGFP* reporter; Fig. 5C), and (iv) appearance of PAs (using the IbpA-msfGFP reporter; Fig. 5D). Next to *tnaA^WT^*, the examined *tnaA* alleles included evolved allele *tnaA^Δ106^* (causing a very high fraction of PA-bearing cells; Fig. 3B), evolved allele *tnaA^259fs^* (causing a low fraction of PA-bearing cells and emerged in heat resistant mutant H17; Fig. 2B), or *tnaA^K270A^* (expressing the catalytically compromised TnaA variant). While the short exposure to tryptophan clearly induced all TnaA variants (Fig. 5A), only induction of TnaA^Δ106^ and TnaA^259fs^ coincided with induction of heat resistance (Fig. 5B), HSPs (Fig. 5C), and PAs (Fig. 5D). In fact, the heat resistance and HSP induction of MG1655 *tnaA^Δ106^* and MG1655 *tnaA^259fs^* mutants could rival those of MG1655 *rpoH^I54T^* in which HSPs and heat resistance are constitutively upregulated in the absence of any obvious protein aggregation (Fig. 5B to D). Additionally, these results also indicate that σ^S^ deficiency is not a requirement for these *tnaA* alleles to confer their protective effect.

**FIG 6:**
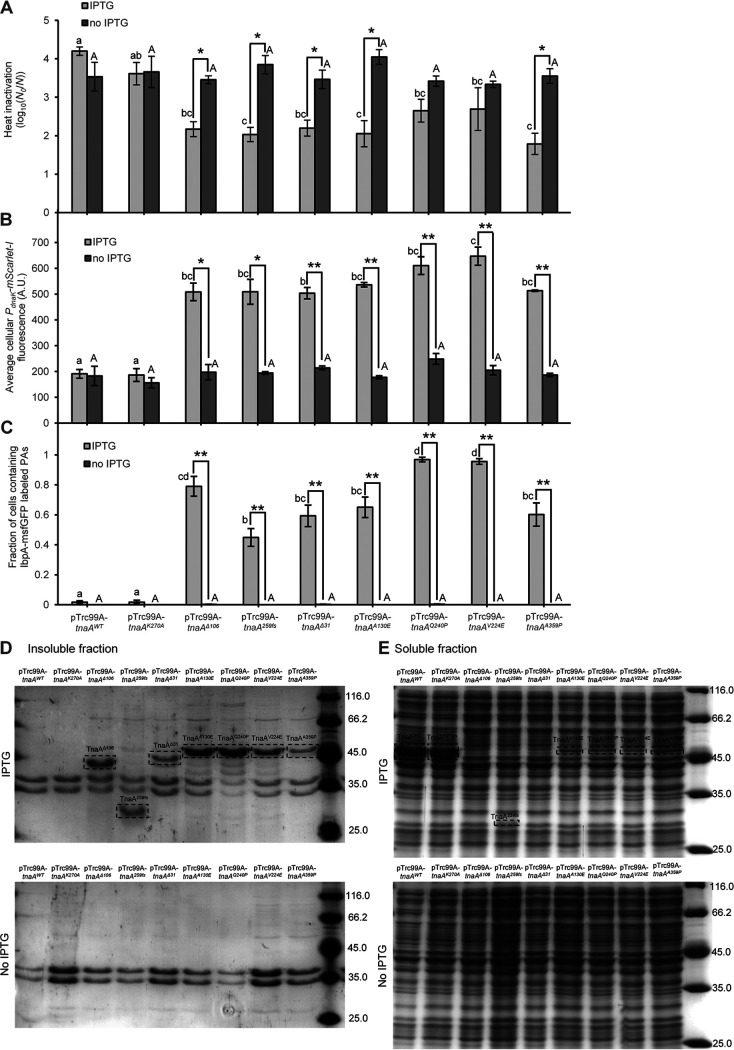
(A) Logarithmic reduction factor after exposure to heat (55°C for 15 min, recovery on TSA) of AB-grown exponential-phase populations of MG1655 Δ*lacY ibpA-msfGFP* P*_dnaK_-mScarlet-I* carrying the indicated pTrc99A-*tnaA* plasmids with (IPTG; light gray) or without (no IPTG; dark gray) IPTG induction. (B) Average cellular mScarlet-I fluorescence of AB-grown exponential-phase populations of MG1655 Δ*lacY ibpA-msfGFP* P*_dnaK_-mScarlet-I* carrying the indicated pTrc99A-*tnaA* plasmids with (IPTG; light gray) or without (no IPTG; dark gray) IPTG induction. The average number of observed cells per strain and condition per independent experiment was 427.3, and sample sizes were always between 59 and 1193 cells. (C) Fraction of cells containing an IbpA-msfGFP labeled PA of AB-grown exponential-phase populations of MG1655 Δ*lacY ibpA-msfGFP* P*_dnaK_-mScarlet-I* carrying the indicated pTrc99A-*tnaA* plasmids with (IPTG; light gray) or without (no IPTG; dark gray) IPTG induction. On average, 95.4 cells were observed per strain and condition per independent experiment, and all sample sizes were between 90 and 108 cells. (D) Representative SDS-PAGE analysis of insoluble protein fractions of AB-grown exponential-phase populations of MG1655 Δ*lacY dnaK-msfGFP* carrying the indicated pTrc99A-*tnaA* plasmids with (top) and without (bottom) IPTG induction. (E) Representative SDS-PAGE analysis of soluble protein fractions of AB-grown exponential-phase populations of MG1655 Δ*lacY dnaK-msfGFP* carrying the indicated pTrc99A-*tnaA* plasmids with (top) and without (bottom) IPTG induction. For panels D and E, numbers in black indicate the molecular weights of the protein ladder bands (in kDa) and the dashed rectangles indicate the bands presumed to correspond to the indicated TnaA variants (for molecular weights, see Table 1). For all panels, strains were grown to exponential phase in AB medium (i.e., 4.5 h of growth after 1/1,000 dilution of stationary-phase cultures) with or without addition of 100 μM IPTG for the last 2 h of growth. For panels A to C, the displayed means were determined over three independent experiments, and the error bars indicate the standard error over these experiments. Asterisks indicate a statistically significant difference for induced compared to noninduced populations of the same strain (Student’s *t* tests followed by Bonferroni correction; *, *P*  ≤ 0.05; **, *P* ≤ 0.01), while different lowercase (for IPTG-induced populations) or capital (for noninduced populations) letters indicate statistically significant differences among strains (Tukey HSD *post hoc* test, *P value* ≤ 0.05).

**FIG 7:**
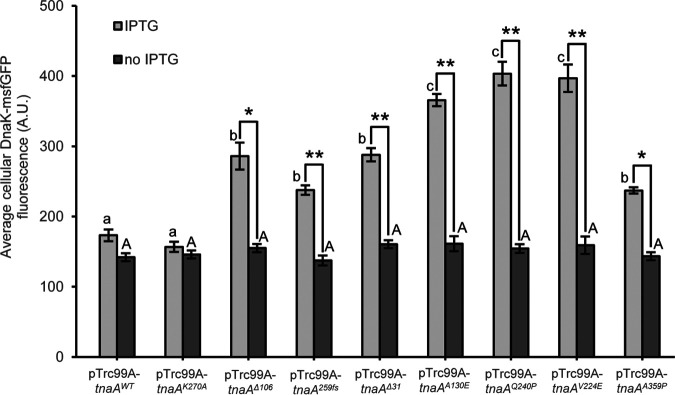
Average cellular msfGFP fluorescence of AB-grown exponential-phase populations of MG1655 Δ*lacY dnaK-msfGFP* carrying the indicated pTrc99A-*tnaA* plasmids with (IPTG; light gray) or without (no IPTG; dark gray) IPTG induction. The average number of observed cells per strain and condition per independent experiment was 296.6, and sample sizes were always between 47 and 1,032 cells. Strains were grown to exponential phase in AB medium (i.e., 4.5 h of growth after 1/1,000 dilution of stationary-phase cultures) with or without addition of 100 μM IPTG for the last 2 h of growth. The displayed means were determined over three independent experiments, and the error bars indicate the standard error over these experiments. Asterisks indicate a statistically significant difference for induced compared to noninduced populations of the same strain (Student’s *t* tests followed by Bonferroni correction; *, *P* ≤ 0.05; **, *P* ≤ 0.01), while different lowercase (for IPTG-induced populations) or capital (for noninduced populations) letters indicate statistically significant differences among strains (Tukey HSD *post hoc* test, *P* value ≤ 0.05).

To independently validate these findings across more of the selected *tnaA* alleles, both control alleles (i.e., *tnaA^WT^* and *tnaA^K270A^*) and a set of heat-selected alleles (i.e., *tnaA^Δ^*^106^, *tnaA^259fs^*, *tnaA^Δ^*^31^, *tnaA^A130E^*, *tnaA^Q240P^*, *tnaA^V224E^*, and *tnaA^A359P^*; Table 1) were individually cloned downstream of the IPTG (isopropyl-β-d-thiogalactopyranoside)-controlled P*_trc_* promoter in the pTrc99A vector and transformed to the MG1655 Δ*lacY ibpA-msfGFP* P*_dnaK_-mScarlet-I* (Fig. 6) and MG1655 Δ*lacY dnaK-msfGFP* (Fig. 7) reporter strains. Exponential-phase cultures of the resulting strains were subsequently induced with IPTG and examined for (i) heat resistance (Fig. 6A), (ii) HSP expression level (via the RpoH-controlled *P_dnaK_-mScarlet-I* reporter in Fig. 6B and the DnaK-msfGFP reporter in Fig. 7), (iii) appearance of PAs (via the IbpA-msfGFP reporter; Fig. 6C), and (iv) PA content (as approached by SDS-PAGE and mass spectrometry analysis of the insoluble and soluble protein content; Fig. 6D and E and Fig. S3). This confirmed that ectopic expression of the heat-selected *tnaA* alleles led to increased heat resistance (Fig. 6A) and increased levels of HSPs (Fig. 6B and Fig. 7) and PAs (Fig. 6C) compared to the *tnaA^WT^* and *tnaA^K270A^* alleles. In two of the heat-selected *tnaA* alleles (*tnaA^Q240P^* and *tnaA^V224E^*), the increase in heat resistance was found not to be statistically significant (Fig. 6A), presumably because plasmid-based overexpression of TnaA^Q240P^ and TnaA^V224E^ coincided with very large amounts of misfolded proteins and PAs (Fig. 6C) that likely imposed a burden on the cell. These experiments also indicate the dominance of the heat-selected *tnaA* alleles over the chromosomal *tnaA^WT^* allele that is still present in the reporter strains, which is in line with our hypothesis that these are gain-of-function alleles.

10.1128/mBio.01129-21.5 FIG S3 SDS-PAGE gel loaded with insoluble protein fractions of AB-grown exponential-phase populations of MG1655 Δ*lacY ibpA-msfGFP* P*_dnaK_-mScarlet-I* carrying the indicated pTrc99A-*tnaA* plasmids. Cultures were 100 μM IPTG-induced for the last 2 h of growth. Bands 1 to 3 were excised from this gel and processed for mass spectrometry analysis. TnaA^Δ106^ (predicted at 40.6 kDa; Table 1) was determined to be the most abundant protein in excised band 1, and TnaA^259fs^ (predicted at 32.2 kDa; Table 1) was found to be the most abundant protein in excised bands 2 and 3. All lanes were run on the same gel. The figure is cropped and composited from a larger image. Numbers in black indicate the molecular weights of the protein ladder bands (in kDa). Download FIG S3, TIF file, 1.9 MB. Copyright © 2021 Mortier et al. 2021 Mortier et al. https://creativecommons.org/licenses/by/4.0/ This content is distributed under the terms of the Creative Commons Attribution 4.0 International license.

Importantly, although the wild-type TnaA protein is known to be a soluble cytoplasmic protein, SDS-PAGE analysis of the insoluble and soluble protein fractions of the above-mentioned plasmid strains clearly revealed that the heat-selected TnaA^Δ106^ TnaA^259fs^, TnaA^Δ31^, TnaA^A130E^, TnaA^Q240P^, TnaA^V224E^, and TnaA^A359P^ variants (in contrast to TnaA^WT^ and TnaA^K270A^) massively ended up in the insoluble protein fraction when their expression was induced with IPTG (Fig. 6D and E), indicating that these variants are themselves aggregation-prone and can thus serve as the direct molecular cause of HSP induction. The abundant presence of TnaA in the insoluble protein fraction was indeed confirmed by mass spectrometry analysis for the strains expressing TnaA^Δ106^ and TnaA^259fs^. TnaA has been identified in the gel bands with a 100% protein probability (Fig. S3, Data Set S1).

### *In silico* analysis of heat-selected TnaA variants.

**FIG 8:**
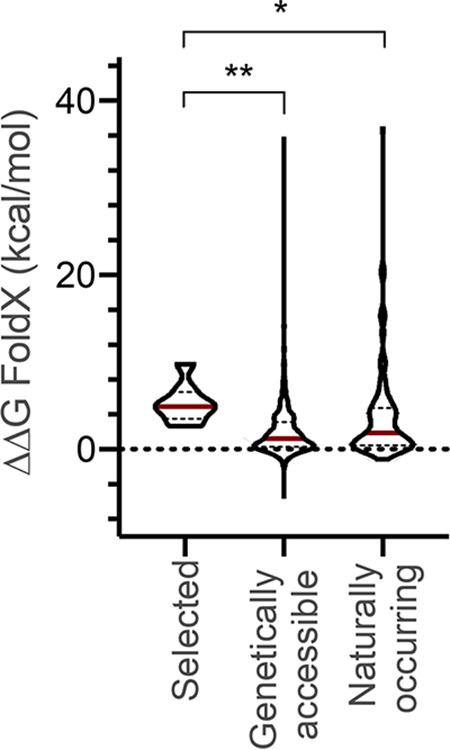
Violin plots of the predicted effects on TnaA stability as calculated through FoldX, using the TnaA pbd-structure 2oqx (57). The stability of heat-selected TnaA variants with single amino acid substitutions is compared to (i) all possible variants that are genetically accessible through a single point mutation and (ii) variants that are found to be naturally occurring in TnaA orthologues. The dashed horizontal line indicates a ΔΔG value of 0 (corresponding to variants with unaltered stability compared to TnaA^WT^). Within each violin plot, median values are indicated by a red horizontal line, while dashed horizontal lines indicate 1st and 3rd quartiles. Significance was determined with a Kruskal-Wallis test with *post hoc* Dunn’s multiple-comparison test (*, *P* ≤ 0.05; **, *P* ≤ 0.01).

Closer examination of the heat-selected full-length TnaA variants indicated that they typically incurred single amino acid substitutions to Glu or Pro (Table 2 and represented in Fig. S4), of which the residues (upon misplacement) were described to be particularly detrimental to overall protein structure (28). In fact, thermodynamic stability calculations (using FoldX [29]) revealed that each of the selected substitutions significantly destabilizes the native structure of the TnaA protein, while the catalytically compromised TnaA^K270A^ variant experiences no significant structural destabilization (ΔΔG scores in Table 2). Moreover, comparing the distribution of the FoldX values of the heat-selected mutants with the distributions obtained either from (i) all TnaA single substitutions that can theoretically be genetically accessed through a single point mutation or (ii) naturally occurring TnaA orthologs strongly suggests heat selection toward TnaA variants with severe structural destabilization (Fig. 8). In addition, the heat-selected substitutions had a zero or extremely low frequency of occurrence in the natural (and likely functional) orthologs (Table 2), further suggesting clear selective pressure toward structural disruption. As such, these findings further underscore that the heat-selected TnaA variants are indeed affected in their folding fidelity and inclined to trigger HSP expression because of this feature.

**TABLE 2:** Characterization of heat-selected TnaA variants harboring a single amino acid substitution^*a*^

Mutation	% MT^*b*^	% WT^*c*^	Occurring AAs in orthologous cluster^*d*^	FoldX ΔΔG (kcal/mol)
Q119P	0.0	36.2	A, G, L, V, I, C, M, Y, D, E, R, K, Q, N, H	3.19
A130E	0.0	32.0	A, L, V, I, S, T, C, F	9.83
F131C	0.0	28.0	A, L, V, I, F, P	4.41
V224E	0.0	50.0	L, V, I, M, F, Y	6.91
V225E	0.0	46.8	L, V, I, C, M, W, F, Y, D, Q, N	5.47
Q240P	0.0	54.6	A, L, V, I, S, T, M, Y, E, R, K, Q, N, H	5.20
K283E	0.5	47.2	A, S, T, D, E, R, K, N, H	2.65
A359P	0.0	89.4	A, G	4.67
K270A	0.0	100.0		0.01

a The catalytically compromised TnaA^K270A^ variant is included in the bottom row for comparison.

b Percentage of entries in the alignment where the mutant residue occurs.

c Percentage of entries where the wild-type residue occurs.

d aa, amino acids.

10.1128/mBio.01129-21.6 FIG S4 Ribbon representation of the TnaA protein structure (pdb-2oqx [57]), with the 8 positions at which the heat-selected single amino acid substitutions occurred highlighted in green surface representation. Download FIG S4, TIF file, 1.8 MB. Copyright © 2021 Mortier et al. 2021 Mortier et al. https://creativecommons.org/licenses/by/4.0/ This content is distributed under the terms of the Creative Commons Attribution 4.0 International license.

### TnaA-PAs also provide epigenetically inheritable longer-term heat resistance.

Time-lapse fluorescence microscopy monitoring of MG1655 cells chromosomally equipped with *tnaA^Δ106^* or *tnaA^259fs^* (and different reporter constructs) after halting tryptophan-mediated induction revealed that over subsequent generations HSP expression (as judged by the *P_htpG_-msfGFP* reporter) homogeneously returned to basal levels (shown for *tnaA^Δ106^* in Fig. S5A). On the other hand, the formed TnaA-PAs (as judged by the IbpA-msfGFP reporter) segregated asymmetrically among sister cells, thereby creating PA-bearing (PA^+^) and PA-lacking (PA^–^) siblings (shown for *tnaA^Δ106^* in Fig. S5B). The homogeneous extinction of HSP expression in an emerging microcolony (despite heterogeneous segregation of PAs) underscores that increased HSP expression is triggered by the initial production of misfolded TnaA variants, and not *per se* by the PA structures into which they assemble. Moreover, the heat resistance of MG1655 *tnaA^Δ106^* and MG1655 *tnaA^259fs^* was similar (Fig. 5B), despite the fact that in MG1655 *tnaA^Δ106^* the TnaA-PAs were more abundant (Fig. 5D), further emphasizing that the size or stability of the PA structures is not necessarily instructive for the level of HSP and heat resistance raised.

10.1128/mBio.01129-21.7 FIG S5 (A) Representative epifluorescence (reporting *P_htpG_* expression) images of MG1655 *P_htpG_-msfGFP tnaA^K270A^* and MG1655 *P_htpG_-msfGFP tnaA^Δ106^* AB-grown exponential-phase founder cells (induced for 2 h with 1.25 mM tryptophan) growing without further induction into microcolonies on AB agarose pads. (B) Representative epifluorescence (reporting IbpA-msfGFP labeled PA localization; indicated by white arrow) images of MG1655 *ibpA-msfGFP tnaA^K270A^* and MG1655 *ibpA-msfGFP tnaA^Δ106^* AB-grown exponential-phase founder cells (induced for 2 h with 1.25 mM tryptophan) growing without further induction into microcolonies on AB agarose pads. Image acquisition was done at 15 and 90 minutes after founder cells were placed on the pad. Scale bars correspond to 2μm. Download FIG S5, TIF file, 2.5 MB. Copyright © 2021 Mortier et al. 2021 Mortier et al. https://creativecommons.org/licenses/by/4.0/ This content is distributed under the terms of the Creative Commons Attribution 4.0 International license.

**FIG 9:**
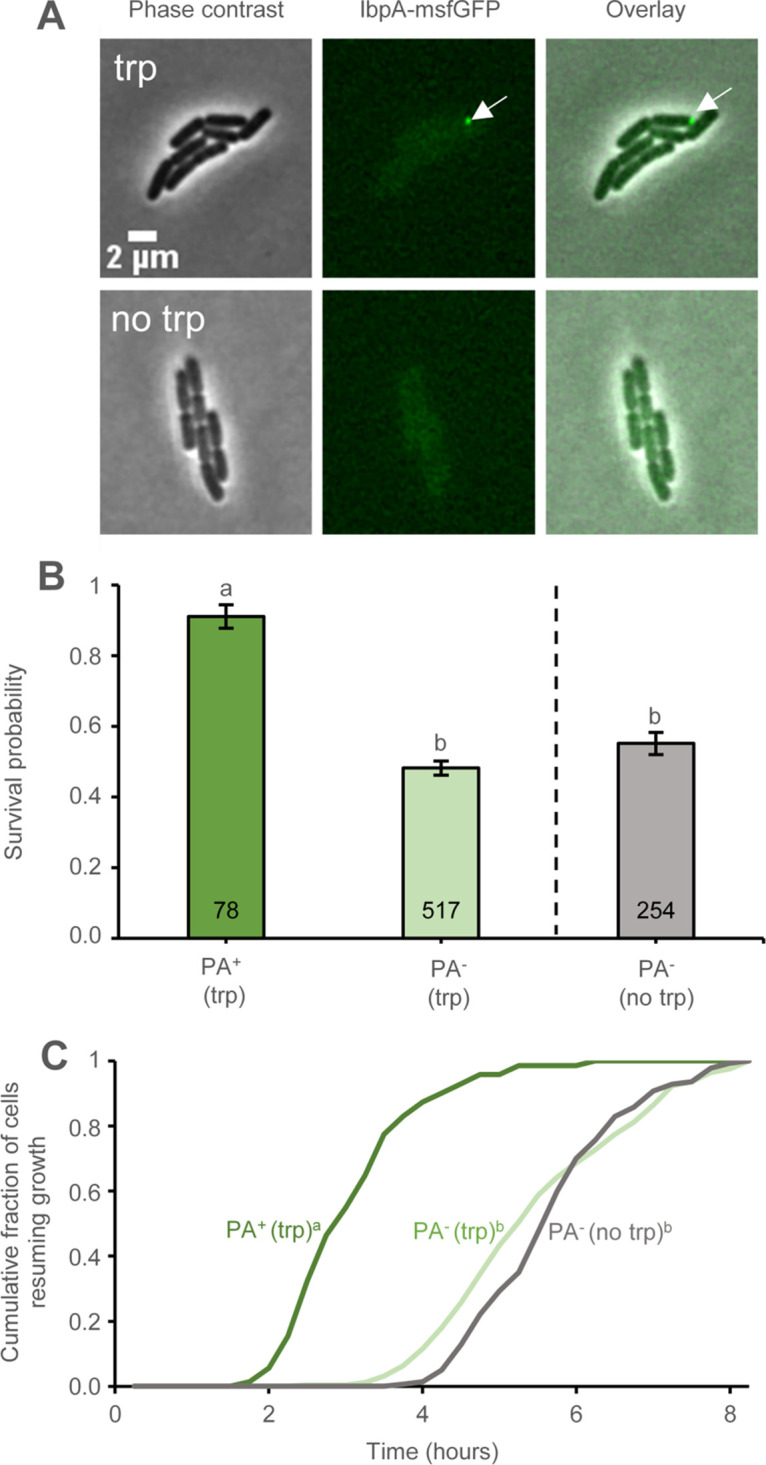
Tryptophan-induced (1 mM for 2 h) and noninduced exponential-phase founder cells of MG1655 *ibpA-msfGFP tnaA^Δ106^* were grown for ca. 3 generations on AB agarose pads in the absence of tryptophan induction. After heat treatment (53.5°C for 15 min) of the resulting microcolonies, survival probability, and lag time of PA-bearing and PA-free cells were determined and compared. (A) Representative phase contrast, epifluorescence (reporting the asymmetrically segregated IbpA-msfGFP labeled PA indicated by the white arrow) and superimposed images of MG1655 *ibpA-msfGFP tnaA^Δ106^* microcolonies resulting from either a tryptophan-induced (trp; upper panel) or noninduced (no-trp; lower panel) founder cell. Scale bar corresponds to 2 μm. (B) Survival probability (i) of PA-bearing (PA^+^; dark green; *n* = 78) and PA-free (PA^–^; light green; *n* = 517) siblings within heat-treated MG1655 *ibpA-msfGFP tnaA^Δ106^* microcolonies stemming from previously induced (trp) founder cells and (ii) of PA-free (PA^–^; gray; *n* = 245) siblings within heat-treated MG1655 *ibpA-msfGFP tnaA^Δ106^* microcolonies stemming from noninduced (no trp) founder cells. Different letters indicate significant differences among the bins according to a generalized linear mixed model with the microcolonies considered random factors (Tukey HSD *post hoc* test, *P* value ≤ 0.05). Error bars indicate bootstrapped estimates of the standard error of the mean fraction of surviving cells. (C) Cumulative resuscitation time distributions (i) of surviving PA-bearing (PA^+^; dark green; *n* = 71) and PA-free (PA^–^; light green; *n* = 249) siblings within heat-treated MG1655 *ibpA-msfGFP tnaA^Δ106^* microcolonies stemming from previously induced (trp) founder cells and (ii) of surviving PA-free (PA^–^; gray; *n* = 140) siblings within heat-treated MG1655 *ibpA-msfGFP tnaA^Δ106^* microcolonies stemming from noninduced (no trp) founder cells. Different letters in superscript indicate significant differences (*P* value ≤ 0.05) among the bins (Kolmogorov-Smirnov tests followed by Bonferroni correction). For panels B and C, data were pooled from three independent experiments.

Nevertheless, we recently documented the occurrence of a different PA-based heat resistance phenomenon (18). In fact, asymmetric inheritance of an ancestral PA (i.e., stemming from a prior heat shock or prior expression of an aggregation-prone protein many generations before) was shown to epigenetically endow the PA^+^ cell with improved heat resistance compared to its PA^–^ siblings (18). In order to examine whether ancestral TnaA-PAs could have such a similar protective effect, well after increased HSP expression coinciding with their original emergence has dampened out, MG1655 *tnaA^Δ106^* was tryptophan-induced for 2 h in a liquid culture and subsequently grown for 2 h on agarose pads lacking tryptophan. The resulting microcolonies, now typically consisting of one PA^+^ and several PA^–^ isogenic siblings (Fig. 9A), were subsequently heat challenged, after which, survival and resuscitation time of both types of cells were compared. This clearly revealed (i) that PA^+^ siblings were endowed with an (on average) 1.9-fold increased survival chance (Fig. 9B), while (ii) the PA^+^ survivors were furthermore endowed with an (on average) 2.5 h decrease in resuscitation time compared to PA^–^ siblings (Fig. 9C). In fact, survival frequency and resuscitation time of PA^–^ siblings were similar to those of MG1655 *tnaA^Δ106^* cells not previously triggered with tryptophan (Fig. 9B and C), indicating that the contribution of the ancestrally elevated HSP levels in the tryptophan-triggered PA^–^ cells meanwhile indeed dampened out completely.

As such, the aggregating TnaA variants cause (i) an immediate population-level heat resistance resulting from the surge in HSP levels and (ii) a secondary subpopulation-level heat resistance in those siblings retaining/inheriting the ancestral TnaA-PA many generations afterward.

## DISCUSSION

Our results essentially indicate that adaptive evolution can capitalize on erosive mutations that alter protein folding fidelity and subsequent aggregation dynamics, thereby enabling cells to autonomously raise HSP expression and boost their robustness against future proteotoxic stresses. This (bottom-up) adaptive evolutionary strategy deviates from common expectations to find (top-down) upregulation of the heat shock response through mutations in its master regulators (such as the dedicated RpoH sigma factor), but instead relies on preemptive self-inflicted damage. Indeed, while the protein quality control network is known to alleviate the impact of a proteotoxic stress by restoring the stability of stress-affected proteins, our findings now (reciprocally) indicate that the acquisition of mutations sacrificing protein folding fidelity of even a single nonessential protein presents an evolutionary mechanism to tune the basal levels and readiness of the chaperone network. More generally, our findings indicate that some loss-of-function mutations might as well be orthogonal gain-of-function mutations, further implying that eroding genes with a degenerating sequence (e.g., pseudogenes that are no longer under stringent selection [30]) are not necessarily on route to being evolutionarily purged and lost from the genome but can actually be actively retained by natural selection.

Interestingly, the reason for the *tnaA* allele being an apparent hot spot for such folding-compromising mutations in our setup is not related to the enzymatic activity of the encoded protein (Fig. 3C and Table 1), despite the fact that indole is otherwise known as a signal molecule with a myriad of physiological effects (e.g., on plasmid stability [31], cell division [32], antibiotic tolerance [33, 34], virulence [35] and biofilm formation [36]). In fact, it rather, seems related to the fact that *tnaA* presents a nonessential (i.e., disposable) gene that becomes fully expressed toward the early stationary phase of TSB-grown cells (i.e., when alleviated catabolite repression and the presence of tryptophan boost the *tnaCAB* operon [23–25]). Since, during our iterative directed evolution regime, heat shocks were consistently administered to TSB-grown stationary-phase cultures, the timing of HSP expression seems to have been optimized by incurring folding-compromising mutations in a gene properly expressed toward this state.

This apparent selection for the proper timing of resistance deployment underscores the possible modularity of this adaptive mechanism. Indeed, incurring folding-compromising mutations in a (nonessential) gene that is only expressed during a particular environmental condition that tends to coincide with proteotoxic stress would now provide leverage to preemptively upregulate HSP expression and cellular robustness under such conditions. Furthermore, in terms of fitness cost, the transient induction of HSPs by targetedly sacrificing the folding of a timely expressed protein might outcompete mechanisms that more constitutively upregulate HSPs via upstream regulatory mutations (e.g., *rpoH* mutants) or a more general attenuation of folding fidelity (e.g., ribosome mutants). In fact, it was most recently shown that synthetically engineered E. coli mutants with increased proteome-wide mistranslation rates (through an error-prone ribosome [37 or depleted levels of cellular tRNA [38]) displayed constitutively upregulated HSP levels and concurrent heat resistance, although indeed, often at the cost of reduced protein synthesis and growth (38). Additionally, mistranslation inherently imposes a proteome-wide burden on the cell to deal with a potentially nonfunctional quasi-species of protein, while limiting that burden to a single protein would naturally be more beneficial.

For some folding-compromised TnaA variants, the specifics of aggregation seem to result in persistent PAs that become asymmetrically inherited for multiple generations (Fig. 9A). This is of interest since PA inheritance itself has most recently been linked to beneficial features in both Saccharomyces cerevisiae (39) and E. coli (18) model systems. In fact, in agreement with our observations regarding heat-induced PAs in E. coli (18), asymmetric cross-generational segregation of ancestral TnaA-PAs leads to a subpopulation of PA^+^ siblings endowed with clearly improved cellular robustness in terms of higher survival frequency and faster resuscitation speed in response to a heat shock (Fig. 9B and C). Unlike cells experiencing protein aggregation, cells inheriting ancestral (i.e., preformed/established) PAs do not display increased expression of HSPs, although their heat resistance might actually stem from coinheritance of PA-associated HSPs that could fortify the PA^+^ cell (18). While the underlying mechanism of this phenomenon is still elusive, this additional (longer-term) benefit of protein aggregation might nevertheless also constitute a supplementary selectable advantage that shaped the actual nature of some TnaA-variants and the stability of the resulting TnaA-PAs. Interestingly, and in contrast to the faster resuscitation displayed in this study, intracellular PAs have recently also been correlated with dormancy and persistence, indicating that we are currently still scratching the surface with regard to PA physiology (8, 40).

Finally, since our data indicate that erosive folding-compromising mutations in proteins can be considered selectable gain-of-function mutations, it is likely that such mutations have left currently unrecognized evolutionary watermarks in the protein sequence space. Indeed, next to their structural or enzymatic cellular function, the evolution of (some) proteins might also have been instructed by their (conditional) folding instability and resulting capacity to boost HSP expression. Likewise, expression patterns of folding-compromised proteins might become tuned to those conditions most in need of higher HSP levels.

In summary, our results reveal a new paradigm in bacterial adaptive physiology in which mutations compromising the folding stability of specific proteins can counterintuitively have a selective advantage because of their subtle upregulating effect on cellular HSP levels. The finding that folding-compromising mutations can actually be positively selected for and are not *per se* loss-of-function mutations can change our view on the evolution of protein sequences.

## MATERIALS AND METHODS

### Bacterial strains and growth conditions.

The bacterial strains and plasmids used in this study are listed in Table S1, and primers are listed in Table S2. Escherichia coli K-12 strain MG1655 was used as the main background throughout this study. For liquid culturing of bacteria, either tryptone soy broth (TSB; Oxoid, Basingstoke, UK), lysogeny broth according to Lennox (LB), or AB medium (supplemented with 10 μg/ml thiamine, 25 μg/ml uracil, and 1% Casamino Acids) was used as indicated. AB medium (with the above-mentioned supplements) was also used as solid medium to make agarose pads intended for time-lapse microscopy by the addition of 2% agarose (Eurogentec, Seraing, Belgium). In the case of single time point microscopy snapshots, 0.85% KCl agarose pads (2% agarose) were used. Incubation for cell growth was always done at 37°C, except for the appropriate times during strain construction when growth at 30°C or 42°C was required. Liquid cultures were incubated aerobically with shaking (250 rpm) in tubes containing 4 ml of medium. Stationary-phase cultures were obtained by ca. 16 h of growth, while exponential-phase cultures were obtained by diluting stationary-phase cultures 1/100 or 1/1,000 in fresh medium and allowing growth for ca. 3 to 5 h (depending on the dilution and the medium used).

10.1128/mBio.01129-21.1 TABLE S1 Overview of strains and plasmids used in this study. Download Table S1, DOCX file, 0.02 MB. Copyright © 2021 Mortier et al. 2021 Mortier et al. https://creativecommons.org/licenses/by/4.0/ This content is distributed under the terms of the Creative Commons Attribution 4.0 International license.

10.1128/mBio.01129-21.2 TABLE S2 Overview of primers used in this work. When relevant, primer attachment sites are indicated in bold, spacer sequences in orange, and artificial ribosome binding sites in purple. In case of the *tna^AK270A^*, the synthetic mutation in the primer is underlined. Download Table S2, DOCX file, 0.01 MB. Copyright © 2021 Mortier et al. 2021 Mortier et al. https://creativecommons.org/licenses/by/4.0/ This content is distributed under the terms of the Creative Commons Attribution 4.0 International license.

When appropriate, the following chemicals were added to the medium at the indicated final concentrations: 100 μg/ml ampicillin (Fisher Scientific, Pittsburgh, PA, USA), 50 μg/ml kanamycin (Applichem, Darmstadt, Germany), 20 μg/ml tetracycline (Applichem), 0.2% l-arabinose (Acros Organics, Geel, Belgium), 1 to 1.25 mM l-tryptophan (Acros Organics), and 100 μM isopropyl β-d-1-thiogalactopyranoside (IPTG; Acros Organics).

### Construction of mutant strains.

Mutant alleles (i.e., *tnaA^K270A^*, *tnaA^Δ^*^106^, *tnaA^259fs^*, and *rpoH^I54T^*) were exchanged with the corresponding MG1655 wild-type alleles using a previously described two-step process of selection and counterselection (41). For the *tnaA^Δ106^* and *tnaA^259fs^* alleles, the gene was first replaced by an amplicon containing the *tetA-sacB* marker prepared on E. coli XTL298 using the primers P1 and P2 (Table S2). In the following step, counterselection against the *tetA-sacB* cassette was used to replace it with a *tnaA* amplicon obtained on the *tnaA* spontaneous mutants (MT3 and H17) using primers P3 and P4 (Table S2). The *tnaA^K270A^* allele was constructed by placing a *tetA-sacB* amplicon (obtained with primers P2 and P6; Table S2) immediately downstream of the 270 codon, and the desired mutation was synthetically incorporated in the upstream primer used to obtain the amplicon for counterselection (P7; Table S2). For the point mutation located in the *rpoH* allele, the *tetA-sacB* cassette (obtained using primers P10 and P11) was inserted downstream of the gene since it is important for proper cell fitness (42, 43), and the clones lacking the cassette after the counterselection step (with an amplicon obtained with primers P12-P13 on a laboratory strain harboring the *rpoH^I54T^* allele [unpublished data]) were screened for the presence of the mutation by sequencing.

The in-frame deletion of *tnaA* was performed according to the method of Datsenko and Wanner (44). Briefly, an amplicon prepared with primers P8 and P9 on pKD13 (containing the kanamycin resistance cassette) was recombineered in-frame after the start codon of the target gene of a pKD46-equipped strain. The kanamycin resistance gene was flanked by *frt* sites, enabling it to be excised by transiently equipping the strain with the plasmid pCP20 (expressing the Flp site-specific recombinase [45]). An in-frame deletion of *lacY* was made in a similar fashion by creating an amplicon on MG1655 *lacY::frt-nptI-frt* (18) using primers P27 and P28.

To construct chromosomal C-terminal transcriptional fusions of *htpG* or *tnaA* with *msfGFP*, the *msfGFP-frt-nptI-frt* cassette was amplified from the previously described pDHL1029 plasmid (46) using primers P15 and P16 (*htpG*) or P3 and P4 (*tnaA*). Similarly, to create a C-terminal transcriptional fusion of *DnaK* with *mScarlet-I*, the *mScarlet-I-frt-nptI-frt* cassette was amplified from pBAM1-Tn*5*-*mScarlet-I* using primers P35 and P36. Subsequent recombineering into the correct locus was achieved by lambda Red-mediated recombination using pKD46 (44). The amplicon was placed 5 bp after the stop codon of the gene of interest, creating an artificial operon and ensuring cotranscription. A strong synthetic ribosome binding site (BBa_B0034; sequence AAAGAGGAGAA [47]) was used to facilitate *msfGFP* or *mScarlet-I* expression. Subsequently, the *frt*-flanked kanamycin resistance cassette was excised by site-specific recombination by transiently equipping the strain with plasmid pCP20. Equipping strains with the previously described chromosomal translational *ibpA*-*msfgfp* fusion (18) was achieved in similar fashion. The amplicon for recombineering was obtained from MG1655 *ibpA-msfGFP-frt-nptI-frt* (18) using primers P19 and P20, followed by the above-mentioned steps to achieve the desired translational fusion.

All constructed mutants were verified by PCR, using primers that anneal upstream and downstream of the engineered locus (Table S2). Subsequently, the mutated genes of interest were confirmed by sequencing (Macrogen, Amsterdam, the Netherlands).

### Construction of plasmids.

A set of pTrc99A plasmids capable of the IPTG-inducible expression of different *tnaA* alleles was constructed by first making an amplicon of the corresponding *tnaA* allele with primer pair P23-P24. These primers amplified the *tnaA* open reading frame and introduced NcoI and XbaI restriction sites to the ends of the amplicon. Subsequently, digestion of both the pTrc99A vector and the amplicons with NcoI and XbaI allowed the directed ligation of the amplicon into the vector.

Plasmid pBAM1-Tn*5*-*mScarlet-I* was constructed by amplifying the pBAM1-Tn*5* backbone from pBAM1-Tn*5*-*mVenus* (48) (using primers P29 and P30) and creating an amplicon of the *mScarlet-I* gene with primers P31 and P32. These amplicons were subsequently assembled into the pBAM1-Tn*5*-*mScarlet-I* plasmid using Gibson assembly (New England BioLabs, Ipswich, MA, USA).

All constructed plasmids were confirmed by amplicon sequencing of the engineered sites (Macrogen) and introduced into the target host strains by electroporation and selection for antibiotic resistance encoded by the plasmid.

### Heat treatment of liquid cultures.

For heat treatment, cultures were harvested by centrifugation (6,000 × *g*, 5 min) and resuspended in an equal volume of 0.85% KCl. Next, 50 μl of the resuspended culture was transferred aseptically to a sterile PCR tube and heat-treated for 15 min in a T3000 thermocycler (Biometra, Göttingen, Germany) at the indicated temperatures. Additionally, unstressed control cultures were kept at room temperature for the duration of the heat treatment. Samples were aseptically retrieved from the PCR tubes and subsequently used to determine survival, as described below.

### Population level determination of viability.

Heat-stressed and unstressed bacterial cultures were serially diluted in 0.85% KCl and subsequently spotted (5 μl) onto tryptone soy agar (TSA; Oxoid) or LB agar (corresponding to the initial growth conditions unless indicated otherwise) as previously described (49). In the case of growth in AB medium, cells were recovered on either TSA or LB agar plates, as indicated. After 24 h of incubation at 37°C, the colonies in spots containing between 5 and 50 colonies were counted to determine the CFU/ml, so that the limit of quantification was 1,000 CFU/ml. Subsequently, the logarithmic reduction factor, log_10_ (*N*_0_/*N*), was determined, in which *N*_0_ and *N* represent the CFU/ml prior to and after heat treatment, respectively.

### Selection of heat-resistant mutants by directed evolution.

Heat-resistant mutants of MG1655 Δ*rpoS ibpA-msfGFP* were obtained by repeatedly subjecting independent overnight TSB cultures to increasingly severe 15-min heat shocks (from 51°C to 55°C with 0.5°C increments) with intermittent resuscitation and outgrowth of the survivors. After each heat shock, the heat-treated samples were diluted 1/100 in fresh TSB and regrown for 23 h at 37°C before the next round of heat treatment. Following nine cycles of selection, a single surviving clone from each culture was purified on TSA and tested for increased heat resistance at 55°C (15 min), as described above. Additionally, a number of independent lineages were subjected to the same regime in the absence of heat stress in order to determine the potential selective effect of serially passing through TSB.

### Whole-genome sequencing (WGS).

Genomic DNA was isolated from overnight LB cultures using the GeneJET genomic DNA purification kit (Thermo Fisher Scientific, Waltham, MA, USA), after which 150- bp paired-end libraries were prepared using the Flex library prep kit (Illumina, San Diego, CA, USA) and the Nextra DNA CD index kit (Illumina). Sequencing was performed with an Illumina MiniSeq sequencer and analyzed with CLC Genomics Workbench (Qiagen, Hilden, Germany). The sequencing reads were trimmed and mapped to the reference genome (MG1655) and analyzed for single nucleotide polymorphisms (SNPs), indels, and structural variants.

### Determining indole concentration.

Indole concentration was determined based on a previously described method (50). Stationary-phase TSB cultures were harvested (6,000 × *g*, 5 min), and the supernatant was transferred to a new recipient, to which 300 μl of Kovac’s reagent (Sigma-Aldrich, Saint Louis, MO, USA) was added. After 2 min of incubation, 20 μl from the top was transferred to a recipient containing 100 μl of 37% HCl and 300 μl of 100% ethanol. The absorbance of 200 μl of this mixture was measured at 550 nm in a microtiter plate using a Multiskan RC instrument (Thermo LabSystems, Vantaa, Finland). Indole concentration was estimated using a calibration curve obtained by the absorbance values at 550 nm after adding 0 to 400 μM indole (Applichem) to stationary-phase cultures of MG1655 Δ*tnaA*.

### Protein extraction.

For protein extraction, 40 ml of an exponential-phase culture was harvested (6,000 × *g*, 10 min, 4°C) and resuspended in 10 mM Tris-HCl (pH 7.5) containing 150 mM NaCl and 5 mM β-mercaptoethanol. The resuspension volume was normalized based on the initial optical density at 600 nm. To break the cells, resuspensions were sonicated (VCX30, Sonics & Materials, Newtown, CT, USA) on ice for a total of 2 min (6 pulses of 20 s at 30% amplitude with 20-s pauses in between). To separate the cellular protein content in soluble and insoluble fractions, the lysate was centrifuged at 13,000 × *g* for 15 min at 4°C. The supernatant was collected and retained as the soluble fraction. The pellet (insoluble fraction) was subsequently washed three times (13,000 × *g* for 15 min at 4°C) with buffer X (50 mM HEPES [pH 7.5], 300 mM NaCl, 5 mM β-mercaptoethanol, 0.1 mM EDTA, and protease inhibitor cocktail [Sigma-Aldrich]). The samples were further purified by three washing steps (13,000 × *g* for 15 min at 4°C) with buffer Y (same as buffer X but without protease inhibitor and supplemented with 0.8% [vol/vol] Triton X_100 and 0.1% sodium deoxycholic acid), with sonicating after every wash step for 10 s (5 pulses of 2 s at 30% amplitude with 2-s pauses in between). Finally, samples were resuspended in buffer Z (50 mM HEPES [pH 7.5], 8.0 M urea), heat-treated (10 min, 90°C), and used for SDS-PAGE (12% gel, 20 μl per well). After SDS-PAGE, gels were stained with Coomassie brilliant blue G-250 as previously described (51).

### Western blotting.

Cells were harvested by centrifugation at 4,000 × *g* for 30 min, washed first with physiological water and then washed with 10 ml of buffer A (50 mM HEPES [pH 7.5], 300 mM NaCl, 5 mM β-mercaptoethanol, and 1.0 mM EDTA). Pellets were finally resuspended in 20 ml of buffer B (buffer A supplemented with a protease inhibitor cocktail). Cells were broken with a Glen Creston cell homogenizer (20,000 to 25,000 lb/in^2^) and additional sonication (Branson digital sonifier 50/60 HZ) on ice with alternating 2-min cycles (15 pulses at 50% power with 30-s pauses on ice, until completing 2 min of total sonication time).

For protein separation, SDS-PAGE was performed using Any kD precast gels (Bio-Rad, Hercules, CA, USA), after which, proteins were transferred on a nitrocellulose membrane (Trans-Blot Turbo Mini 0.2-μm nitrocellulose transfer packs; Bio-Rad) with the Trans-Blot Turbo transfer system (Bio-Rad). Afterward, the membrane was incubated for 1 h in 5% nonfat dry milk in TBST (Tris-buffered saline with Tween 20, pH 8.0) and then stained overnight with the primary antibodies, i.e., monoclonal mouse anti-DnaK (1 μg/ml; Aviva Systems Biology, San Diego, CA) or anti-GroEL (1 μg/ml; Abcam, Cambridge, UK). The membranes were washed three times for 10 min with 0.5% Tween 20 in phosphate-buffered saline (PBS) buffer and stained with the secondary antibody for 2 h (goat anti-mouse IgG HRP; Abcam). After washing, horseradish peroxidase (HRP) was detected with an enhanced chemiluminescence system (ChemiDoc Imaging System; Bio-Rad), and band intensity was quantified with the Image Lab software (Bio-Rad). The density of each band after background subtraction was expressed in fold change compared to the parental Δ*rpoS* strain. Western blot (WB) analysis was performed four times on four independent stationary-phase cultures of each strain.

### Liquid chromatography and mass spectrometry (LC-MS/MS).

For protein identification, gel pieces were extracted based on the protocol of Shevchenko et al. (52). Digestion was performed overnight at 37°C. Samples were desalted using Pierce C18 solid-phase extraction columns according to the manufacturer’s instructions (Thermo Scientific) and dried in a SpeedVac until dry and dissolved in 20 μl 5% acetonitrile (ACN) and 0.1% formic acid. The digested and desalted samples were diluted 10 times and injected (5 μl) and separated on an Ultimate 3000 UPLC system (Dionex, Thermo Scientific) equipped with an Acclaim PepMap100 precolumn (C_18_ particle size, 3 μm; pore size, 100 Å; diameter, 0.075 mm; length, 20 mm; Thermo Scientific) and a C_18_ PepMap rapid-separation liquid chromatography (RSLC) system (particle size, 2 μm; pore size, 100 Å; diameter, 50 μm; length, 150 mm; Thermo Scientific) using a linear gradient (0.300 μl/min). The composition of buffer A is pure water containing 0.1% formic acid. The composition of buffer B is pure water containing 0.08% formic acid and 80% acetonitrile. The fraction 0 to 4% of buffer B (80% ACN, 0.08% formic acid) increased from 0 to 4% in 3 min, from 4 to 10% B in 12 min, from 10 to 35% in 20 min, from 35 to 65% in 5 min, and from 65 to 95% in 1 min and stayed at 95% for 10 min. The fraction of buffer B, decreased from 95 to 5% in 1 min and stayed at 5% for 10 min. The Orbitrap Elite mass spectrometer (Thermo Scientific) was operated in positive ion mode with a nanospray voltage of 2.1 kV and a source temperature of 250°C. Pierce LTQ Velos ESI positive ion calibration mix (catalog no. 88323, Thermo Scientific) was used as an external calibrant. The instrument was operated in data-dependent acquisition mode with a survey MS scan at a resolution of 70,000 (full width at half maximum [fwhm]) for the mass range of *m/z* 400 to 1,600 for precursor ions, followed by MS/MS scans of the top 10 most intense peaks with +2, +3, +4, and +5 charged ions above a threshold ion count of 16,0001e+6 at 17,500 resolution (full width at half maximum [fwhm]) using a normalized collision energy of 25 eV with an isolation window of 3.0 *m/z*, Apex trigger of 5 to 15 s, and dynamic exclusion of 10 s. All data were acquired with Xcalibur 3.1.66.10 software (Thermo Scientific).

### Protein identification.

Tandem mass spectra were extracted using Progenesis v 4.1 (Nonlinear Dynamics, UK). All MS/MS samples were analyzed using Mascot v 2.2.06 (Matrix Science, London, UK) and X! Tandem v X! (The Global Proteome Machine [GPM], thegpm.org; Tandem Alanine [2017.2.1.4]). Mascot was set up to search the uniprot_ecolimax_ database (282,225 entries, where the mutated sequences have been added) assuming the digestion enzyme trypsin. X!, Tandem was set up to search a reverse concatenated uniprot_ecolimax_ database (unknown version, 564,448 entries). Mascot and X! Tandem were searched with a fragment ion mass tolerance of 0.20 Da and a parent ion tolerance of 12 ppm and 2 possible miscleavages. Carbamidomethyl of cysteine was specified in Mascot and X! Tandem as a fixed modification. Deamidation of asparagine and glutamine and oxidation of methionine were specified in Mascot as variable modifications. Glu->pyro-Glu of the N terminus, ammonia-loss of the N terminus, gln->pyro-Glu of the N terminus, deamidation of asparagine, and glutamine and oxidation of methionine were specified in X! Tandem as variable modifications.

Scaffold v 4.11.0 (Proteome Software, Inc., Portland, OR) was used to validate MS/MS-based peptide and protein identifications. Peptide identifications were accepted if they could be established at greater than 97.0% probability by the Peptide Prophet algorithm (53) with Scaffold delta-mass correction. Protein probability was assigned by the Protein Prophet algorithm (54).

### Time-lapse fluorescence microscopy (TLFM).

For TLFM, appropriate dilutions of cell cultures were transferred to agarose pads containing the appropriate medium on a microscopy slide and covered with a cover glass attached to a 125-μl Gene Frame (Thermo Fisher Scientific) to hold the cover glass on the microscopy slide. TLFM was performed on a Ti-Eclipse inverted microscope (Nikon, Champigny-sur-Marne, France) equipped with a ×60 Plan Apo λ oil objective, a TI-CT-E motorized condenser, and a Nikon DS-Qi2 camera. Green fluorescent protein (GFP) was imaged using a quad-edge dichroic (395/470/550/640 nm) and a fluorescein isothiocyanate (FITC) single emission filter. A SpecraX LED illuminator (Lumencor, Beaverton, OR, USA) was used as the light source, using the 470/24 excitation filter. Temperature was controlled with an cage incubator (Okolab, Ottaviano, Italy).

Images were acquired using NIS-Elements software (Nikon), and the resulting pictures were further handled with the open source software ImageJ. The average cellular fluorescence of cells was determined using the open source software Ilastik (55), which was trained to robustly identify and segment bacterial cells and exclude debris and out-of-focus cells. Background fluorescence was subtracted using NIS-elements software.

### Single-cell determination of viability and resuscitation time.

Cells were placed on agarose pads on a microscopy slide, as described above, and monitored with TLFM during growth for 2 h at 37°C (approximately 3 generations). Subsequently, the XY coordinates of the observed microcolonies were determined with NIS-Elements software, and the slide was subjected to a semilethal heat shock by taping the slide to the lid of a thermocycler set to the appropriate temperature (53.5°C, 15 min). After the heat shock, the locations of the heat-exposed microcolonies were traced back using the XY coordinates, and the cells were further monitored for 8 h to determine survival and lag time. Subsequently, the number of cells surviving the heat shock in microcolonies was determined by monitoring cells with TLFM. Cells were marked as surviving cells when they were observed to grow and divide within 8 h after the heat treatment. The time of the first binary fission after heat treatment was used as a proxy for resuscitation time. Cells were binned according to whether they had an inclusion body by visual determination or a fluorescent IbpA-msfGFP focus before heat treatment.

### Thermodynamic stability calculations of TnaA variants.

Based on the TnaA sequence of the E. coli K-12 MG1655 strain used throughout this study (protein and nucleotide sequences in Data Set S2), all possible single amino acid substitutions that can theoretically result from a single nucleotide mutation were generated, resulting in a list of 2,765 variants. In addition, naturally occurring substitutions were gathered from a set of sequences orthologous to E. coli K-12 TnaA from the OMA (Orthologous Matrix) database (56), resulting in 218 sequences after removing outliers with internal indels over 10 residues compared to the reference sequence (Data Set S3). A substitution was classified as naturally occurring if it occurs in at least one of the TnaA orthologues. The effects of these substitutions and the heat-selected substitutions on TnaA thermodynamic stability were calculated and compared using FoldX3.0 with default settings (29) on the pdb-structure 2oqx (57).

10.1128/mBio.01129-21.8 Data Set S1: Peptide composition and probabilities of TnaA for the different gel bands shown in Fig. S3. Peptide probability is expressed in likelihood with 1 corresponding to 100% confidence. Download Data Set S1, XLSX file, 0.01 MB. Copyright © 2021 Mortier et al. 2021 Mortier et al. https://creativecommons.org/licenses/by/4.0/ This content is distributed under the terms of the Creative Commons Attribution 4.0 International license.

10.1128/mBio.01129-21.9 Data Set S2: Wild-type *tnaA* nucleotide sequence (top) and TnaA protein sequence (bottom) of the E. coli K-12 MG1655 strain used in this study. Download Data Set S2, DOCX file, 0.01 MB. Copyright © 2021 Mortier et al. 2021 Mortier et al. https://creativecommons.org/licenses/by/4.0/ This content is distributed under the terms of the Creative Commons Attribution 4.0 International license.

10.1128/mBio.01129-21.10 Data Set S3: (1) TnaA ortholog alignment and natural amino acid occurrence. (Top) alignment of a set of 218 TnaA orthologs retrieved from the OMA (Orthologous Matrix) database (56) with the TnaA protein sequence from MG1655 (ECOLX03116). Column A shows the bacterial strain as per the OMA database nomenclature. Columns C to RE show the amino acid in each position as aligned to the MG1655 reference sequence. (Bottom) Occurrences of each amino acid per position in the alignment. Positions were numbered with respect to the reference sequence (TnaA from MG1655). (2) FoldX results per mutation. Effects of exhaustive amino acid mutations on the stability of the MG1665 TnaA protein as predicted by the FoldX forcefield (29) using the PDB structure 2oqx (57). Mutations are identified in column A as (wild-type amino acid – chain in PDB structure – position in chain – mutant amino acid). For example, mutation KA5A denotes a mutation of Lys in position 5 of chain A to an Ala. Column B (ΔΔG [kcal/mol]) shows the average free energy difference calculated by FoldX over three repeats of the prediction. Column C (SD [kcal/mol]) gives the standard deviation of the free energy difference over three repeats of the prediction. Column D (original aa), column E (mutant aa), and column F (position) repeat the wild-type amino acid, the mutant amino acid, and the position in the chain, respectively. Download Data Set S3, XLSX file, 0.9 MB. Copyright © 2021 Mortier et al. 2021 Mortier et al. https://creativecommons.org/licenses/by/4.0/ This content is distributed under the terms of the Creative Commons Attribution 4.0 International license.

### Statistical analysis.

Statistical analyses (analysis of variance [ANOVA], Tukey honestly significant difference [HSD] *post hoc* test, *t* test, Kolmogorov-Smirnov (KS) test, generalized linear mixed models, Bonferroni correction, bootstrapping [and the appropriate tests to test for underlying assumptions]), were carried out using the open source software R (R Core Team, 2020) (60). Differences were regarded as significant when the *P* value was ≤0.05. Means and the corresponding standard errors were typically calculated over three independent experiments.

To estimate the standard error of the surviving cellular fractions in Fig. 9B, the original sample size was bootstrapped (sampled with replacement) 10,000 times to calculate the mean fractions of surviving cells. This allowed the calculation of a bootstrapped estimation of the standard error of the mean fraction of surviving cells by determining the standard deviation of the bootstrapped means.
