# Erratum for Mortier and Gayán et al., “Gene Erosion Can Lead to Gain-of-Function Alleles That Contribute to Bacterial Fitness”

**DOI:** 10.1128/mBio.02467-21

**Published:** 2021-09-28

**Authors:** Julien Mortier, Elisa Gayán, Ronald Van Eyken, Oscar Enrique Torres Montaguth, Ladan Khodaparast, Laleh Khodaparast, Bert Houben, Sebastien Carpentier, Frederic Rousseau, Joost Schymkowitz, Abram Aertsen

**Affiliations:** a KU Leuven, Department of Microbial and Molecular Systems, Leuven, Belgium; b Switch Laboratory, KU Leuven, Department of Cellular and Molecular Medicine, Leuven, Belgium; c VIB, Center for Brain & Disease Research, Leuven, Belgium; d KU Leuven, SYBIOMA: Facility for Systems Biology Mass Spectrometry, Leuven, Belgium

## ERRATUM

Volume 12, no. 4, e01129-21, 2021, https://doi.org/10.1128/mBio.01129-21. For the authors Ladan Khodaparast, Laleh Khodaparast, Bert Houben, Frederic Rousseau, and Joost Schymkowitz, the affiliation “VIB, Center for Brain & Disease Research, Leuven, Belgium” should be included as shown above.

