# Supplementary figures and images for: Gene Erosion Can Lead to Gain-of-Function Alleles That Contribute to Bacterial Fitness
Source: mBio. 2021 Jul 6;12(4):e01129-21. doi: 10.1128/mBio.01129-21 (PMC8406189; doi:10.1128/mBio.01129-21)

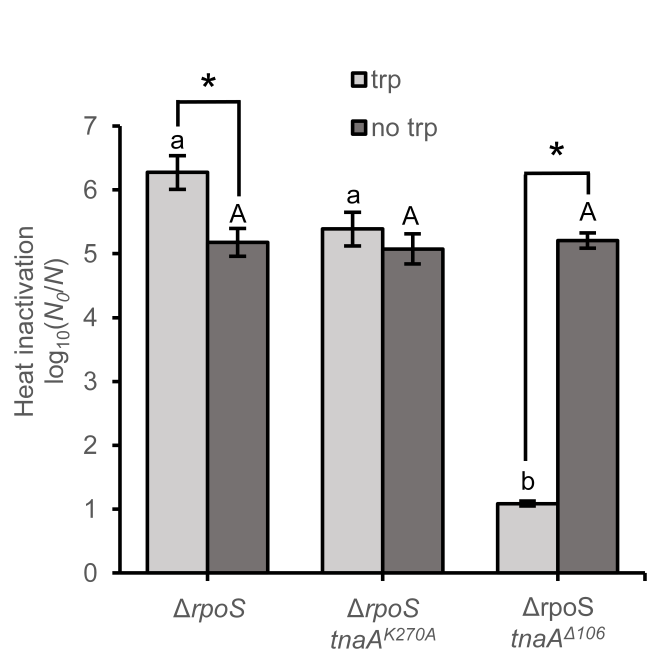

Supplement: FIG S1 [file mbio.01129-21-sf001.tif]

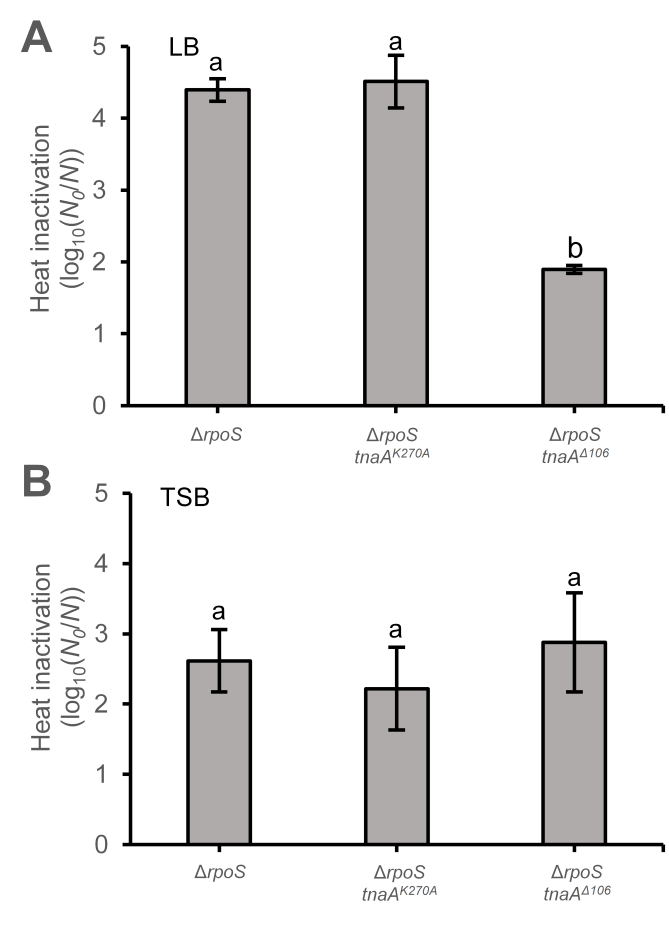

Supplement: FIG S2 [file mbio.01129-21-sf002.tif]

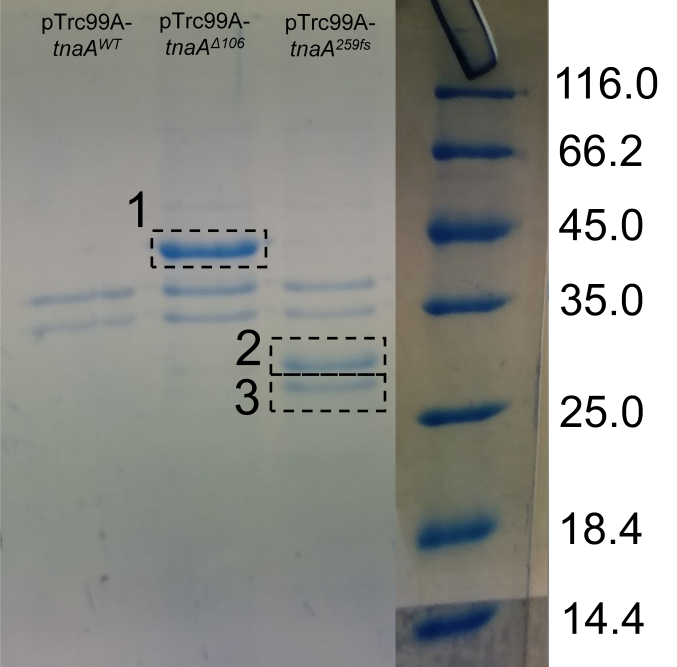

Supplement: FIG S3 [file mbio.01129-21-sf003.tif]

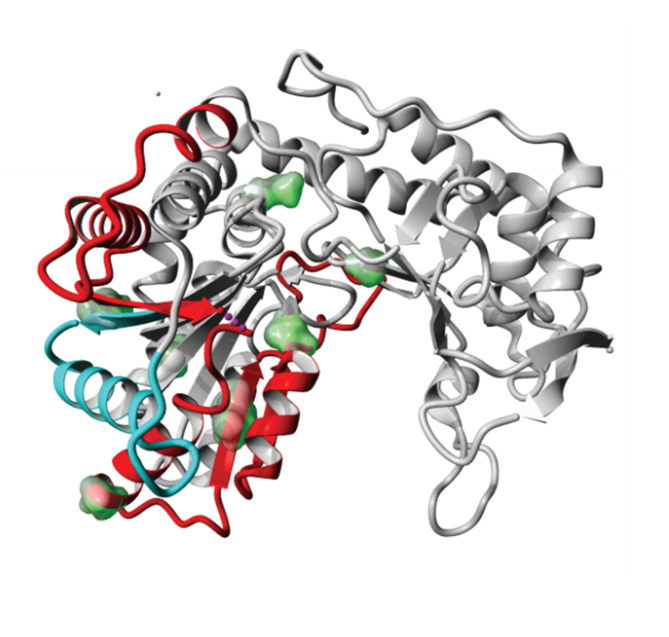

Supplement: FIG S4 [file mbio.01129-21-sf004.tif]

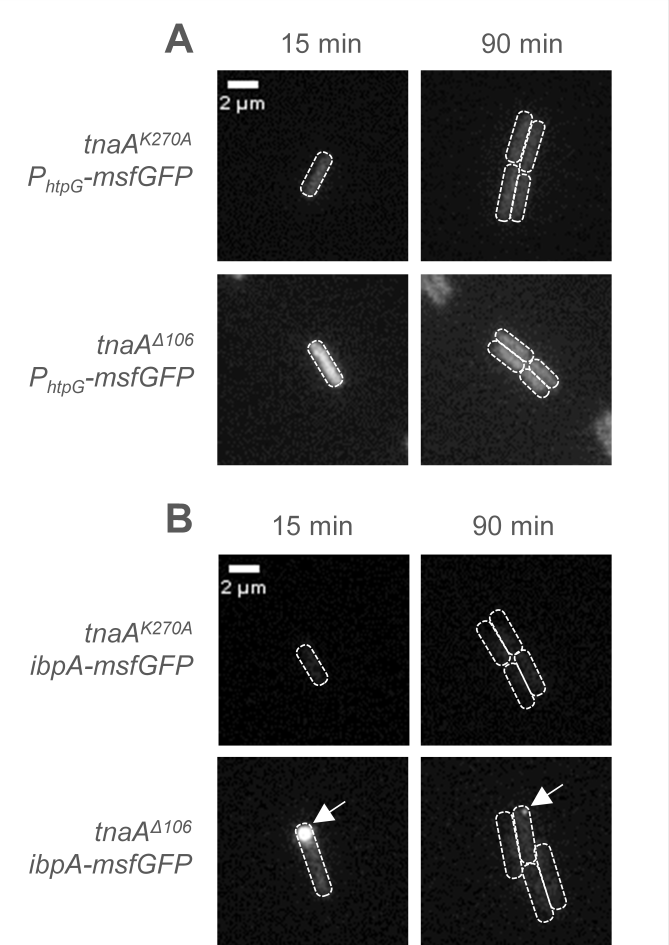

Supplement: FIG S5 [file mbio.01129-21-sf005.tif]
