# Supplementary material for: Gene Erosion Can Lead to Gain-of-Function Alleles That Contribute to Bacterial Fitness
Source: mBio. 2021 Jul 6;12(4):e01129-21. doi: 10.1128/mBio.01129-21 (PMC8406189; doi:10.1128/mBio.01129-21)
Supplement: TABLE S1 [file mbio.01129-21-st001.docx]

| **Strain** | **Description** | **Source or reference** |
| --- | --- | --- |
| MG1655 | *E. coli* MG1655 wild type | ^58^ |
| MG1655 Δ*rpoS* | MG1655 with an in-frame deletion of *rpoS* Parental strain for heat cycling | ^19^ |
| MTH1 | Heat resistant derivative of MG1655 Δ*rpoS* | This study |
| MT2 | Heat resistant derivative of MG1655 Δ*rpoS* | ^19^ |
| MT3 | Heat resistant derivative of MG1655 Δ*rpoS* | ^19^ |
| MT5 | Heat resistant derivative of MG1655 Δ*rpoS* | ^19^ |
| XTL298 | For preparation of the tetA-sacB amplicon | ^41^ |
| MG1655 Δ*rpoS* Δ*tnaA* | MG1655 with an in-frame deletion of *rpoS* and an in-frame deletion of *tnaA* | This study |
| MG1655 Δ*rpoS* *tnaA^Δ106^* | MG1655 with an in-frame deletion of *rpoS* and the MT3 *tnaA^Δ106^* allele | This study |
| MG1655 Δ*rpoS tnaA^K270A^* | MG1655 with an in-frame deletion of *rpoS* and a K270A mutation in the catalytic site of *tnaA* | This study |
| MG1655 Δ*rpoS* *ibpA-msfGFP* | MG1655 *ΔrpoS* encoding a C-terminal translational fusion of IbpA to msfGFP  Parental strain for heat cycling | This study |
| H1 | Heat resistant derivative of MG1655 Δ*rpoS ibpA-msfGFP* | This study |
| H2 | Heat resistant derivative of MG1655 Δ*rpoS ibpA-msfGFP* | This study |
| H3 | Heat resistant derivative of MG1655 Δ*rpoS ibpA-msfGFP* | This study |
| H4 | Heat resistant derivative of MG1655 Δ*rpoS ibpA-msfGFP* | This study |
| H5 | Heat resistant derivative of MG1655 Δ*rpoS ibpA-msfGFP* | This study |
| H6 | Heat resistant derivative of MG1655 Δ*rpoS ibpA-msfGFP* | This study |
| H7 | Heat resistant derivative of MG1655 Δ*rpoS ibpA-msfGFP* | This study |
| H8 | Heat resistant derivative of MG1655 Δ*rpoS ibpA-msfGFP* | This study |
| H9 | Heat resistant derivative of MG1655 Δ*rpoS ibpA-msfGFP* | This study |
| H10 | Heat resistant derivative of MG1655 Δ*rpoS ibpA-msfGFP* | This study |
| H11 | Heat resistant derivative of MG1655 Δ*rpoS ibpA-msfGFP* | This study |
| H12 | Heat resistant derivative of MG1655 Δ*rpoS ibpA-msfGFP* | This study |
| H13 | Heat resistant derivative of MG1655 Δ*rpoS ibpA-msfGFP* | This study |
| H14 | Heat resistant derivative of MG1655 Δ*rpoS ibpA-msfGFP* | This study |
| H15 | Heat resistant derivative of MG1655 Δ*rpoS ibpA-msfGFP* | This study |
| H16 | Heat resistant derivative of MG1655 Δ*rpoS ibpA-msfGFP* | This study |
| H17 | Heat resistant derivative of MG1655 Δ*rpoS ibpA-msfGFP* | This study |
| UC1^1^ | Unstressed derivative of MG1655 Δ*rpoS ibpA-msfGFP* | This study |
| UC2^1^ | Unstressed derivative of MG1655 Δ*rpoS ibpA-msfGFP* | This study |
| MG1655 Δ*tnaA* | MG1655 with an in-frame deletion of *tnaA* | This study |
| MG1655 *tnaA^K270A^* | MG1655 with a K270A mutation in the catalytic site of *tnaA* | This study |
| MG1655 *tnaA^Δ106^* | MG1655 with the MT3 *tnaA^Δ106^* allele | This study |
| MG1655 *tnaA^259fs^* | MG1655 with the H17 *tnaA^259fs^* allele | This study |
| MG1655 *rpoH^I54T^* | MG1655 with the LMM1010 *rpoH^I54T^* allele | This study |
| MG1655 *ibpA-msfGFP-frt-nptI-frt* | MG1655 encoding a C-terminal unflipped translational fusion of IbpA to msfGFP | ^18^ |
| MG1655 *ibpA-msfGFP* | MG1655 encoding a C-terminal translational fusion of IbpA to msfGFP | ^18^ |
| MG1655 *ibpA-msfGFP tnaA^K270A^* | MG1655 encoding a C-terminal translational fusion of IbpA to msfGFP and containing the *tnaA^K270A^* allele | This study |
| MG1655 *ibpA-msfGFP tnaA^Δ106^* | MG1655 encoding a C-terminal translational fusion of IbpA to msfGFP and containing the MT3 *tnaA* allele | This study |
| MG1655 *ibpA-msfGFP tnaA^259fs^* | MG1655 encoding a C-terminal translational fusion of IbpA to msfGFP and containing the H17 *tnaA^259fs^* allele | This study |
| MG1655 *ibpA-msfGFP rpoH^I54T^* | MG1655 encoding a C-terminal translational fusion of IbpA to msfGFP and containing the LMM1010 *rpoH^I54T^* allele | This study |
| MG1655 *P_htpG_-msfGFP* | MG1655 encoding a transcriptional fusion of *htpG* to msfGFP | This study |
| MG1655 *P_htpG_-msfGFP tnaA^K270A^* | MG1655 encoding a transcriptional fusion of *htpG* to msfGFP and containing the *tnaA^K270A^* allele | This study |
| MG1655 *P_htpG_-msfGFP tnaA^Δ106^* | MG1655 encoding a transcriptional fusion of *htpG* to msfGFP and containing the MT3 *tnaA^Δ106^* allele | This study |
| MG1655 *P_htpG_-msfGFP tnaA^259fs^* | MG1655 encoding a transcriptional fusion of *htpG* to msfGFP and containing the H17 *tnaA^259fs^* allele | This study |
| MG1655 *P_htpG_-msfGFP rpoH^I54T^* | MG1655 encoding a transcriptional fusion of *htpG* to msfGFP and containing the LMM1010 *rpoH^I54T^* allele | This study |
| MG1655 *P_tnaCAB_-msfGFP* | MG1655 encoding a transcriptional fusion of *tnaA* to msfGFP with the wild type *tnaA* allele | This study |
| MG1655 *P_tnaCAB_-msfGFP*  *tnaA^K270A^* | MG1655 encoding a transcriptional fusion of *tnaA* to msfGFP with the *tnaA^K270A^* allele | This study |
| MG1655 *P_tnaCAB_-msfGFP*  *tnaA^Δ106^* | MG1655 encoding a transcriptional fusion of *tnaA* to msfGFP with the MT3 *tnaA^Δ106^* allele | This study |
| MG1655 *P_tnaCAB_-msfGFP*  *tnaA^259fs^* | MG1655 encoding a transcriptional fusion of *tnaA* to msfGFP with the H17 *tnaA^259fs^* allele | This study |
| MG1655 *P_tnaCAB_-msfGFP rpoH^I54T^* | MG1655 encoding a transcriptional fusion of *tnaA* to msfGFP and containing the LMM1010 *rpoH^I54T^* allele | This study |
| MG1655 *lacY::frt-nptI-frt* | MG1655 in which the *lacY* open reading frame is replaced with an *frt*-flanked *nptI* cassette | ^18^ |
| MG1655 Δ*lacY ibpA-msfGFP P_dnaK_-mScarlet-I* | MG1655 carrying an in-frame deletion of *lacY* and encoding a transcriptional fusion of *dnaK* to mScarlet-I and a C-terminal translational fusion of IbpA to msfGFP | This study |
| MG1655 Δ*lacY dnaK-msfgfp* | MG1655 carrying an in-frame deletion of *lacY* and encoding a C-terminal translational fusion of DnaK to msfGFP | ^18^ |
| **Plasmid** | **Description** | **Source or reference** |
| pCP20 | Encodes Flp for recombining *frt* sites (to excise sequences in between) | ^45^ |
| pKD46 | Encodes lambda *red* genes under control of an arabinose-inducible promoter | ^44^ |
| pDHL1029 | Harbors *msfGFP-frt-nptI-frt* site for construction of  msfGFP translational or transcriptional fusions | ^46^ |
| pTrc99A | pTrc99A expression vector carrying the IPTG-inducible P*_trc_* promoter | ^59^ |
| pTrc99A-*tnaA^WT^* | pTrc99A-derived vector for IPTG-inducible expression of the *tnaA^WT^* allele | This study |
| pTrc99A-*tnaA^K270A^* | pTrc99A-derived vector for IPTG-inducible expression of the *tnaA^K270A^* allele | This study |
| pTrc99A-*tnaA^Δ106^* | pTrc99A-derived vector for IPTG-inducible expression of the *tnaA^Δ106^* allele | This study |
| pTrc99A-*tnaA^259fs^* | pTrc99A-derived vector for IPTG-inducible expression of the *tnaA^259fs^* allele | This study |
| pTrc99A-*tnaA^Δ31^* | pTrc99A-derived vector for IPTG-inducible expression of the *tnaA^Δ31^* allele | This study |
| pTrc99A-*tnaA^A130E^* | pTrc99A-derived vector for IPTG-inducible expression of the *tnaA^A130E^* allele | This study |
| pTrc99A-*tnaA^Q240P^* | pTrc99A-derived vector for IPTG-inducible expression of the *tnaA^Q240P^* allele | This study |
| pTrc99A-*tnaA^V224E^* | pTrc99A-derived vector for IPTG-inducible expression of the *tnaA^V224E^* allele | This study |
| pTrc99A-*tnaA^A359P^* | pTrc99A-derived vector for IPTG-inducible expression of the *tnaA^A359P^* allele | This study |
| pBAM1-Tn*5*-*mVenus* | Contains Tn5-*mVenus* transposon | ^48^ |
| pBAM1-Tn*5*-*mScarlet-I* | Contains *mScarlet-I* followed by an *frt-nptI-frt* cassette | This study |
|  |  |  |

UC1 and UC2 were cycled without being subjected to heat stress and are genetically identical to the parental MG1655 Δ*rpoS* *ibpA*-*msfGFP* strain.
