# Supplementary material for: Gene Erosion Can Lead to Gain-of-Function Alleles That Contribute to Bacterial Fitness
Source: mBio. 2021 Jul 6;12(4):e01129-21. doi: 10.1128/mBio.01129-21 (PMC8406189; doi:10.1128/mBio.01129-21)
Supplement: TABLE S2 [file mbio.01129-21-st002.docx]

| **Name** | **Sequence** | **Description** | |  |
| --- | --- | --- | --- | --- |
| P1 | ACATCCTTATAGCCACTCTGTAGTATTAATTAAACTTCTTTAAGTTTTGC**TCCTAATTTTTGTTGACACTCTATC** | | Amplification of the *tetA-sacB* cassette from XTL298 for replacing the *tnaA* allele | |
| P2 | AATATTCACAGGGATCACTGTAATTAAAATAAATGAAGGATTATGTAATG**ATCAAAGGGAAAACTGTCCATATGC** | | Amplification of the *tetA-sacB* cassette from XTL298 for replacing the *tnaA* allele | |
| P3 | GTGTCTTGCGAGGATAAGTGCA | | Control and sequencing of *tnaA* replacement | |
| P4 | CACCGGCAAGATCAACAGGT | | Control and sequencing of *tnaA* replacement | |
| P5 | GCGGTGACGCAGAGCATGCAG​ | | Sequencing of *tnaA* gene | |
| P6 | CCCGCGAAACCTACAAATATGCCGATATGCTGGCGATGTCCGCCAAGAAA**TCCTAATTTTTGTTGACACTCTATC** | | Amplification of the *tetA-sacB* cassette from XTL298 for constructing the *tnaA^K270A^* allele | |
| P7 | CCTACAAATATGCCGATATGCTGGCGATGTCCGCCAAGGCAGATGCGATGGTGCCGATG | | Amplicon for replacing the *tetA-sacB* cassette for constructing the *tnaA^K270A^* allele | |
| P8 | AATATTCACAGGGATCACTGTAATTAAAATAAATGAAGGATTATGTAATG**ATTCCGGGGATCCGTCGACC** | | Amplification of the *frt-nptII-frt* cassette from pKD13 for deleting the *tnaA* gene | |
| P9 | ACATCCTTATAGCCACTCTGTAGTATTAATTAAACTTCTTTAAGTTTTGC**TGTAGGCTGGAGCTGCTTCG** | | Amplification of the *frt-nptII-frt* cassette from pKD13 for deleting the *tnaA* gene | |
| P10 | TGTAATAATCAATTTCCCCTCCGGCAAAACGCCAATCCCCACGCAGATTG**TCCTAATTTTTGTTGACACTCTATC** | | Amplification of the *tetA-sacB* cassette from XTL298 for its insertion downstream of the *rpoH* gene | |
| P11 | TATTTCGTTTTATGCATGACTACCCGTGCTTTAGCAGCATACTCTGCTAA**ATCAAAGGGAAAACTGTCCATATGC** | | Amplification of the *tetA-sacB* cassette from XTL298 for its insertion downstream of the *rpoH* gene | |
| P12 | ATGCCTGCTATTGCTGCTGG | | Control and sequencing of *rpoH* replacement | |
| P13 | TGTTGCCTTTAATGCCGCCTT | | Control and sequencing of *rpoH* replacement | |
| P14 | TGCAAAGTTTAGCTTTAGCC | | Sequencing of *rpoH* gene | |
| P15 | CCGAACCTGTTTATTCGTCGTATGAACCAGCTGCTGGTTTCCTGATGTAAAAAGAGGAGAATACTAGATG**AGTAAAGGTGAAGAACTGTTCACCGG** | | For construction of a transcriptional fusion of *htpG* with *msfGFP* | |
| P16 | GTGCAGATGATGAAAAGAAA AATGCCGGATGACACGAAGGTCATCCGGCA**ATTCCGGGGATCCGTCGACC** | | For construction of a transcriptional fusion of *htpG* with *msfGFP* | |
| P17 | GAAAGTATTGCGTCACTTCACCGCAAAACTTAAAGAAGTTTAAAAAGAGGAGAATACTAGATG**AGTAAAGGTGAAGAACTGTTCAC** | | For construction of a transcriptional fusion of *tnaA* with *msfGFP* | |
| P18 | TGTAGGGTAAGAGAGTGGCTAACATCCTTATAGCCACTCTGTAGTATTA**AATTCCGGGGATCCGTCGACC**​ | | For construction of a transcriptional fusion of *tnaA* with *msfGFP* | |
| P19 | CTGACCTGTCCATTGTGGAAGG | | Control and sequencing of *ibpA* locus | |
| P20 | CTTCTAAGAAGCGAGTAAGTACCTGC | | Control and sequencing of *ibpA* locus | |
| P21 | CCACGGTTGAAGGTGAAG | | Control and sequencing of *htpG* locus | |
| P22 | ACGATACCACCAACAGGC | | Control and sequencing of *htpG* locus | |
| P23 | GTACCCATGGAAAACTTTAAACATCTCCCTG | | For introduction of *tnaA* alleles in pTrc99A | |
| P24 | GCACTCTAGATAACATCCTTATAGCCACTCTGTAG | | For introduction of *tnaA* alleles in pTrc99A | |
| P25 | GTTCTGGCAAATATTCTGAAATGAGCTG | | Control and sequencing of pTrc99A constructions | |
| P26 | GCTTCTGCGTTCTGATTTAATCTG | | Control and sequencing of pTrc99A constructions | |
| P27 | CGACTTCCAGTTCAACATCAG | | Construction of in-frame *lacY* deletions | |
| P28 | GGATGCGAGTGATTAAACTCATAC | | Construction of in-frame *lacY* deletions | |
| P29 | gctgtacaagtaa**GCATGGATGAGCTCTACAAAG** | | Amplification of pBAM1 backbone for construction of pBAM1-Tn*5*-*mScarlet-I* | |
| P30 | ctagtattctcctctttta**AGATGTGTATAAGAGACAGCTGG** | | Amplification of pBAM1 backbone for construction of pBAM1-Tn*5*-*mScarlet-I* | |
| P31 | atacacatcttaaaagaggagaatactag**ATGGTGAGCAAGGGC** | | Amplification of *mScarlet-I* for construction of pBAM1-Tn*5*-*mScarlet-I* | |
| P32 | tcatccatgctta**CTTGTACAGCTCGTCCATG** | | Amplification of *mScarlet-I* for construction of pBAM1-Tn*5*-*mScarlet-I* | |
| P33 | gaacacttaacggctgacatg | | Control and sequencing of pBAM1-Tn*5*-*mScarlet-I* | |
| P34 | atacgcgccttcctgc | | Control and sequencing of pBAM1-Tn*5*-*mScarlet-I* | |
| P35 | GATaGTTGTCGACGCTGAATTTGAAGAAGTCAAAGACAAAAAATAATCGCC**AAAGAGGAGAATACTAGATGGTGAGC** | | For construction of a transcriptional fusion of *dnaK* with *mScarlet-I* | |
| P36 | CGGAGAGGAAATTCCCCTTCGCCCGTGTCAGTATAATTACCCGTTTATA**GGCCGCACCTGCAG** | | For construction of a transcriptional fusion of *dnaK* with *mScarlet-I* | |
| P37 | GCTGTCAATTTTACGTCTTGTC | | Control and sequencing of *dnaK* locus | |
| P38 | CACCCTGGTTACGGTCC | | Control and sequencing of *dnaK* locus | |
|  |  | |  | |
