# Supplementary material for: Gene Erosion Can Lead to Gain-of-Function Alleles That Contribute to Bacterial Fitness
Source: mBio. 2021 Jul 6;12(4):e01129-21. doi: 10.1128/mBio.01129-21 (PMC8406189; doi:10.1128/mBio.01129-21)
Supplement: Data Set S2: [file mbio.01129-21-sd002.docx]

***tnaA* nucleotide sequence of the *E. coli* K12 MG1655 strain used in this study**

ATGGAAAACTTTAAACATCTCCCTGAACCGTTCCGCATTCGTGTTATTGAGCCAGTAAAACGTACCACTCGCGCTTATCGTGAAGAGGCAATTATTAAATCCGGTATGAACCCGTTCCTGCTGGATAGCGAAGATGTTTTTATCGATTTACTGACCGACAGCGGCACCGGGGCGGTGACGCAGAGCATGCAGGCTGCGATGATGCGCGGCGACGAAGCCTACAGCGGCAGTCGTAGCTACTATGCGTTAGCCGAGTCAGTGAAAAATATCTTTGGTTATCAATACACCATTCCGACTCACCAGGGCCGTGGCGCAGAGCAAATCTATATTCCGGTACTGATTAAAAAACGCGAGCAGGAAAAAGGCCTGGATCGCAGCAAAATGGTGGCGTTCTCTAACTATTTCTTTGATACCACGCAGGGCCATAGCCAGATCAACGGCTGTACCGTGCGTAACGTCTATATCAAAGAAGCCTTCGATACGGGCGTGCGTTACGACTTTAAAGGCAACTTTGACCTTGAGGGATTAGAACGCGGTATTGAAGAAGTTGGTCCGAATAACGTGCCGTATATCGTTGCAACCATCACCAGTAACTCTGCAGGTGGTCAGCCGGTTTCACTGGCAAACTTAAAAGCGATGTACAGCATCGCGAAGAAATACGATATTCCGGTGGTAATGGACTCCGCGCGCTTTGCTGAAAACGCCTATTTCATCAAGCAGCGTGAAGCAGAATACAAAGACTGGACCATCGAGCAGATCACCCGCGAAACCTACAAATATGCCGATATGCTGGCGATGTCCGCCAAGAAAGATGCGATGGTGCCGATGGGCGGCCTGCTGTGCATGAAAGACGACAGCTTCTTTGATGTGTACACCGAGTGCAGAACCCTTTGCGTGGTGCAGGAAGGCTTCCCGACATATGGCGGCCTGGAAGGCGGCGCGATGGAGCGTCTGGCGGTAGGTCTGTATGACGGCATGAATCTCGACTGGCTGGCTTATCGTATCGCGCAGGTACAGTATCTGGTCGATGGTCTGGAAGAGATTGGCGTTGTCTGCCAGCAGGCGGGCGGTCACGCGGCATTCGTTGATGCCGGTAAACTGTTGCCGCATATCCCGGCAGACCAGTTCCCGGCACAGGCGCTGGCCTGCGAGCTGTATAAAGTCGCCGGTATCCGTGCGGTAGAAATTGGCTCTTTCCTGTTAGGCCGCGATCCGAAAACCGGTAAACAACTGCCATGCCCGGCTGAACTGCTGCGTTTAACCATTCCGCGCGCAACATATACTCAAACACATATGGACTTCATTATTGAAGCCTTTAAACATGTGAAAGAGAACGCGGCGAATATTAAAGGATTAACCTTTACGTACGAACCGAAAGTATTGCGTCACTTCACCGCAAAACTTAAAGAAGTTTAA

**TnaA protein sequence of the *E. coli* K12 MG1655 strain used in this study.**

MENFKHLPEPFRIRVIEPVKRTTRAYREEAIIKSGMNPFLLDSEDVFIDLLTDSGTGAVTQSMQAAMMRGDEAYSGSRSYYALAESVKNIFGYQYTIPTHQGRGAEQIYIPVLIKKREQEKGLDRSKMVAFSNYFFDTTQGHSQINGCTVRNVYIKEAFDTGVRYDFKGNFDLEGLERGIEEVGPNNVPYIVATITSNSAGGQPVSLANLKAMYSIAKKYDIPVVMDSARFAENAYFIKQREAEYKDWTIEQITRETYKYADMLAMSAKKDAMVPMGGLLCMKDDSFFDVYTECRTLCVVQEGFPTYGGLEGGAMERLAVGLYDGMNLDWLAYRIAQVQYLVDGLEEIGVVCQQAGGHAAFVDAGKLLPHIPADQFPAQALACELYKVAGIRAVEIGSFLLGRDPKTGKQLPCPAELLRLTIPRATYTQTHMDFIIEAFKHVKENAANIKGLTFTYEPKVLRHFTAKLKEV
